# Relaxation damage control via fatigue-hydraulic fracturing in granitic rock as inferred from laboratory-, mine-, and field-scale experiments

**DOI:** 10.1038/s41598-021-86094-5

**Published:** 2021-03-24

**Authors:** Arno Zang, Günter Zimmermann, Hannes Hofmann, Peter Niemz, Kwang Yeom Kim, Melvin Diaz, Li Zhuang, Jeoung Seok Yoon

**Affiliations:** 1 Helmholtz Centre Potsdam GFZ German Research Centre for Geosciences, Telegrafenberg, 14473 Potsdam, Germany; 2 Institute of Geosciences, University of Potsdam, 14469 Potsdam, Germany; 3 Korea Maritime and Ocean University, Busan, Republic of Korea; 4 Korea Institute of Civil Engineering and Building Technology, Goyang, Republic of Korea; 5 DynaFrax UG (Limited), Helmholtzstr. 6, 14467 Potsdam, Germany

**Keywords:** Solid Earth sciences, Energy science and technology

## Abstract

The ability to control induced seismicity in energy technologies such as geothermal heat and shale gas is an important factor in improving the safety and reducing the seismic hazard of reservoirs. As fracture propagation can be unavoidable during energy extraction, we propose a new approach that optimises the radiated seismicity and hydraulic energy during fluid injection by using cyclic- and pulse-pumping schemes. We use data from laboratory-, mine-, and field-scale injection experiments performed in granitic rock and observe that both the seismic energy and the permeability-enhancement process strongly depend on the injection style and rock type. Replacing constant-flow-rate schemes with cyclic pulse injections with variable flow rates (1) lowers the breakdown pressure, (2) modifies the magnitude-frequency distribution of seismic events, and (3) has a fundamental impact on the resulting fracture pattern. The concept of fatigue hydraulic fracturing serves as a possible explanation for such rock behaviour by making use of depressurisation phases to relax crack-tip stresses. During hydraulic fatigue, a significant portion of the hydraulic energy is converted into rock damage and fracturing. This finding may have significant implications for managing the economic and physical risks posed to communities affected by fluid-injection-induced seismicity.

## Introduction

Successfully utilising unconventional energy resources relies critically on understanding and controlling the mechanical deformations of fractured rock mass in the Earth’s upper crust. Examples of such utilisation include creating and sustaining fracture networks in enhanced geothermal systems (EGS) as well as in unconventional oil- and gas reservoirs. Each of these subsurface technologies is governed by the intrinsic properties of fractured rock and its response to primary, natural stresses (lithostatic, tectonic) as well as to secondary, applied stresses (hydraulic stimulation) at a variety of scales. Understanding the mechanisms that control the fracture nucleus and growth is particularly relevant in such complex stress conditions^1^. The principles of fracture growth in the presence of large-scale anisotropic discontinuous rock mass must be studied by observing deformation and fluid flow in mine-scale underground tests^2–6^, which requires instrumenting, monitoring, and interpreting rock-mass behaviour in situ. The fracture architecture, instrument choice, and sensor resolution all affect the overall result of the seismic and aseismic signals that are captured^7,8^. Zang et al.^9^ proposed an iterative process of optimising the necessary observations by combining both underground- and laboratory tests. This process allows a variety of fluid-injection schemes to be applied in the laboratory before testing the most promising schemes in several in-situ experiments in the same rock type.

Human activity perturbs subsurface stresses, thereby causing fractures to become unstable, to propagate, and to coalesce, as documented in induced seismicity^10,11^. This induced seismicity results from various individual causes^12,13^, such as wastewater disposal^14,15^, hydraulic fracturing^16,17^, carbon capture and storage^18,19^, and geothermal operations^20,21^. Although the radiated seismic energy represents only a small fraction of the pumped-in hydraulic energy, perceptions of induced seismic events caused by human operations in the Earth’s subsurface have led to the termination of energy projects^22–25^. The primary goal of energy projects is therefore to manage subsurface operations without the occurrence of seismic events of economic concern^26^. While many scientific articles have reported on induced seismicity^11,12,14,23,27^, few have dealt with suggestions of how to mitigate and reduce fluid-induced seismicity^9^. One option is to use seismic traffic-light systems, which are widely accepted as a risk-mitigation procedure in hydraulic treatment^28,29^. Many concepts have been proposed^30^, but few such systems have actually been applied in the field^31–34^. In these traffic-light systems, fluid injection is stopped, either the treatment pressure is reduced, or the well is shut in or flowed back if certain thresholds of seismic magnitudes are exceeded during injection^35^. Magnitude thresholds are also used when refined advanced and adaptive traffic-light systems are applied^36^. Another option for controlling injection-induced seismicity is to modify the seismic-event distribution via the injection style^17,37^. Using hydro-mechanical-coupled numerical simulations of a naturally fractured geothermal reservoir with Soultz-sous-Forets properties, Yoon et al.^38^ demonstrated that compared with a monotonic injection, a cyclic injection of fluid has the capacity to lower the number of larger-magnitude seismic events while increasing the overall number of smaller events.

This work aims to assess the process of hydraulic-fracture growth during injection experiments in light of both associated induced seismicity and hydro-mechanical parameters, such as formation-breakdown pressure (FBP) and fracture-permeability evolution. For this purpose, we present rich experimental datasets consisting of (1) results from a decametre-scale in-situ test at Äspö Hard Rock Laboratory (HRL) in Sweden performed in June 2015 at a depth of 410 m and (2) results from laboratory hydraulic-fracture tests under triaxial and true-triaxial stress conditions performed at the Korea Institute of Civil Engineering and Building Technology (KICT) from 2016 to 2020. Both datasets are compared with a field-stimulation treatment at an EGS site performed in 2017^39^. While the injection rate^40^, the cumulative injection volume^41,42^, and the injection pressure^43^ have been demonstrated to impact the likelihood of associated induced seismic events, a systematic cross-scale study that investigates the interrelation of fracture growth, seismicity, and permeability enhancement has not yet been performed. In this study, we investigate innovative injection schemes that use cyclic- and pulse-pumping protocols—so-called hydraulic-fatigue tests—to optimise the fracture-growth process. Optimisation includes the analysis of fracturing and re-fracturing stages, acoustic-emission hypocentres and their magnitude-frequency distributions, and fracture-pattern- and related permeability improvement. We find that breakdown pressure decreases with an increasing number of injection cycles, particularly in laboratory testing with hundreds of cycles as well as in the mine. In all scales, seismic *b*-values determined from magnitude-frequency distributions indicate a trend towards larger values in hydraulic fatigue compared with results from conventional tests using monotonic-fluid-pressure injection. This finding reveals that a safer treatment that can mitigate larger seismic events indeed exists. Resulting fracture patterns are quantified with X-ray CT and microscopic inspection in the laboratory and are investigated via impression-packer analysis and seismic-event-hypocentre tracking in the mine. Compared with monotonic-injection tests, laboratory-fatigue tests reveal a more-complex fracture pattern resulting from branching, which is mainly caused by stress release at the fracture tips (cyclic injection) and by rock chips being removed from fracture walls (pulse injection). Decreasing breakdown pressure and seismicity by creating a broader damage zone is in line with our concept of hydraulic fatigue, which is expanded upon in the supplementary material. This concept is also supported by the energy budget analysed in conventional and fatigue tests across scales. In addition to engineering elements that control fluid-injection-fracture growth in rock (breakdown pressure, induced seismicity, permeability), we also investigate the energy budget of the fracture-growth process. In this work, we compute individual energy terms, such as seismically radiated-, hydraulic-, and fracture energy, and estimate the deformation energy from stimulated volumes of rock. Although the absolute energy values differ by orders of magnitude from scale to scale, a scale-independent tendency for a lower ratio of radiated seismic energy to exist with respect to hydraulic energy is documented in the fatigue test as compared with in monotonic injection.

## Hydraulic-fatigue experiments

**Figure 1:**
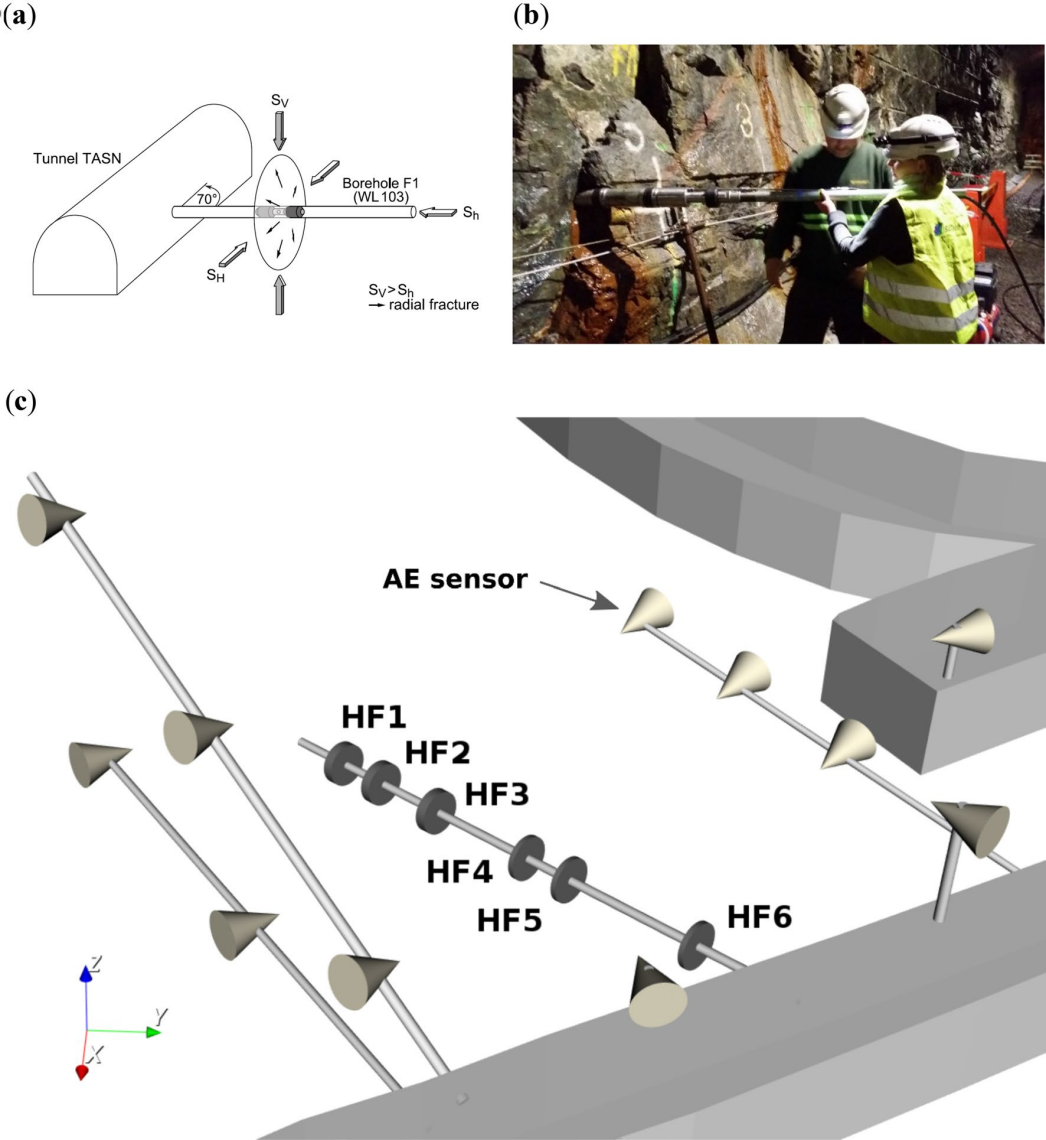
Hydraulic-fracture design at depth of 410 m at Äspö HRL, Sweden. (**a**) A hydraulic-testing borehole (diameter: 102 mm; length: 28 m) is diamond-drilled subparallel to the minimum horizontal stress. The hydraulic fracture (*disk*) opens perpendicular to the minimum principal stress and rapidly grows in the plane of intermediate (vertical) and maximum principal stress (horizontal). (**b**) Photograph of pulse-fatigue hydraulic-fracturing-packer system as it is inserted into the horizontal-injection borehole. (**c**) Geometry of injection borehole with six injection intervals (HF1–HF6) and inclined monitoring boreholes (diameter: 76 mm; length: up to 30 m), which are equipped with 70-kHz AE sensors (*cones*).

A naturally fractured granitic rock cube with a side length of 30 m was monitored at the mine scale during hydraulic-fracturing tests at a depth of 410 m in a Swedish hard-rock^2^. Six hydraulic fractures were propagated from a 28-m-long horizontal borehole that served as an injection borehole in the cube centre. Three injection styles including conventional and fatigue hydraulic fracturing (e.g. cyclic and pulse progressive fracturing) were tested in situ (Fig. 1). Stress conditions at depth indicated maximum horizontal stress as maximum principal stress. The minimum and intermediate principal stresses were oriented sub-horizontally and sub-vertically. The magnitude of maximum principal stress (22 MPa) was about double the value of the minimum and intermediate principal stresses (11–12 MPa)^44^. An injection borehole with a diameter of 102 mm was drilled from the TASN tunnel in the direction of minimum horizontal stress (Fig. 1a). Figure 1b displays the hydraulic-fatigue packer system with a short mandrel for pulsing being inserted into the injection borehole^45^. All injection tests were monitored by an extensive acoustic-emission- (AE), seismic- (geophone, broadband sensor), and electromagnetic-sensor (EME, MT) network. In Fig. 1c, the geometry of the high-frequency monitoring array is shown with eleven AE sensors (70 kHz) located in the monitoring boreholes and the nearby tunnel roofs (Fig. 1c, *cones*). In the testing borehole, pressures were monitored in injection intervals that were free of pre-existing fractures (Fig. 1c, HF1–HF6). Fracture inspection was carried out by combining core logs, the impression packer, and the borehole results. A televiewer tool was used to map the injection borehole before and after hydraulic testing. Upon completing the fracturing and re-fracturing stages, the borehole wall was mapped with an impression packer. The shape of the fractures and their extensions were reconstructed via the impression-packer results and the AE-hypocentre-tracking results^46^. The fracture aperture was computed via the extension of the AE cloud and the measured hydraulic back-flow values. The evolution of permeability was calculated from decline-pressure curves after each injection stage—that is, during shut-in—while taking into account the superposition principle and assuming an infinitely acting radial flow^47,48^:

1 $$k=\frac{ q \mu }{4 \pi h \Delta p} ln\left(\frac{{{\mathrm{t}}}_{0} +\Delta {\mathrm{t}}}{\Delta {\mathrm{t}}}\right),$$

with h = interval length, q = flow rate, µ = dynamic viscosity of the fluid, t_0_ = injection time, Δt = shut-in time, Δp = pressure difference, and ln = natural logarithm.

Hydraulic-fracturing intervals from tests HF1, HF2, and HF3 are located in Ävrö granodiorite (AG) in the deeper part of the hydraulic-testing borehole, HF4 and HF5 intervals are located in fine-grained diorite-gabbro (fgDG), and the HF6 interval is situated in fine-grained granite (fgG) at a distance of 5 m from the tunnel wall (Fig. 1c).

At the laboratory scale, water-injection experiments were performed on Pocheon granite under triaxial^49^ and true-triaxial stress conditions^50^ at KICT. We used true triaxial loading equipment, which is capable of performing hydraulic-fracturing tests while controlling either the injection rate or the pressurisation rate. Samples were cut into cubes with a side length of 100 mm. Hydraulic fractures were propagated from an injection borehole with a diameter of 5 mm using six different injection schemes. The applied stresses were prescribed at 4 MPa, 6 MPa, and 3 MPa for the vertical-, maximum horizontal-, and minimum horizontal stress, respectively. A well-described sample of Jurassic granite from the Pocheon quarry, South Korea, with three distinct planes of weakness (so-called rift, grain, and hardway) was used throughout the tests^49^. The first set of injection-rate-control tests comprised constant-rate-continuous, stepwise-rate continuous, and cyclic-progressive injection schemes analogous to the mine tests. The second set of pressurisation-rate control tests included stepwise-, stepwise pulse-, and cyclic pulse pressurisation. A total of 20 tests were carried out using tap water as injection fluid. Unlike the field test, AE activity was monitored with an array of eight *nano* sensors, two of which directly attached to each lateral face of the sample, thereby leaving the top and bottom of the cube blank. A high-vacuum grease-coupling agent enhanced the contact between the sensors and rock surface. The lateral loading plates had small notches on the lower-left- and upper-right corners to allow space for the sensors. The sensors were fixed with bolts to prevent them from dislodging during the tests. The AE sensors (125–750 kHz) and the data-acquisition system were manufactured and developed by the Physical Acoustic Corporation (MISTRAS Group Inc., Princeton, USA). The AE signals were pre-amplified with a gain of 40 dB. During the tests, injection pressure and AE were monitored. Subsequently, the injectivity of the fractured granite samples was measured via water-injection tests at six different injection rates that had been carefully selected to avoid any further fracturing and ranged from 5–30 mm^3^/s. As a result, an approximately linear relationship was obtained between the injection rate and the plateau of the injection pressure that corresponded with each injection rate. The injectivity was estimated from the slope of this linear relationship.

The cyclic-stimulation concept was first applied at the field scale in August 2017 at the Pohang EGS site in South Korea^39^. In Pohang, a ~ 160 °C granitic geothermal reservoir with low permeability was accessed with two > 4-km-deep wells, PX-1 and PX-2, with a spacing of ~ 600 m at reservoir depth. “Cyclic soft-stimulation treatment”^33,39^ in August 2017 in PX-1 was performed after a conventional stimulation with continuous fluid injection from December 2016 to January 2017^51^. The treatment design was based on the previously described laboratory- and mine-scale experiments and adapted for the site-specific conditions^33^. Three additional stimulations, which are not discussed in this manuscript, were performed in the second well (PX-2) before and after the two PX-1 stimulations reported here.

## Results

### Breakdown pressure and injection-induced seismicity

**Figure 2:**
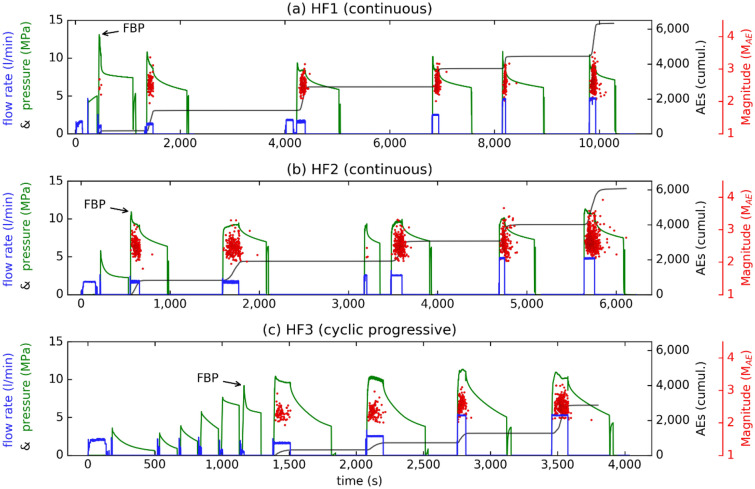
Results of three hydraulic-fracturing tests in Ävrö granodiorite at mine scale. HF1 (**a**) and HF2 (**b**) are conventional fracturing- and re-fracturing tests, respectively, with continuous water injection. HF3 (**c**) is a fatigue test with cyclic, progressive fluid injection before the occurrence of breakdown pressure. Left ordinate indicates flow rate (*blue*) and fluid pressure (*green*). Right ordinate shows cumulative number of AEs (*black curve*) and their magnitudes (*red dots*) over time (s) in the stimulation.

Figure 2 displays results from three hydraulic-fracturing tests in the deeper part of the injection borehole at Äspö HRL inside Ävrö granodiorite (see Fig. 1c, HF1–HF3). The plotted hydraulic parameters are the interval pressure and the flow rate in the injection interval (Fig. 2, left axis). The plotted induced-seismicity parameters are the cumulative number and magnitude of the AE events (Fig. 2, black curve and red dots) obtained from the continuous-recording system^46^. The upper-two panels in Fig. 2 display conventional tests with a monotonic-pressure increase (HF1, HF2), and the lower panel shows the fatigue test with cyclic-progressive fluid injection (HF3). The first pressure increase in each experiment resulted from an integrity test, which was stopped before the formation-breakdown pressure (FBP) had been reached. (FBP is the pressure at which a fracture begins to propagate from the wellbore into the formation).

**Figure 3:**
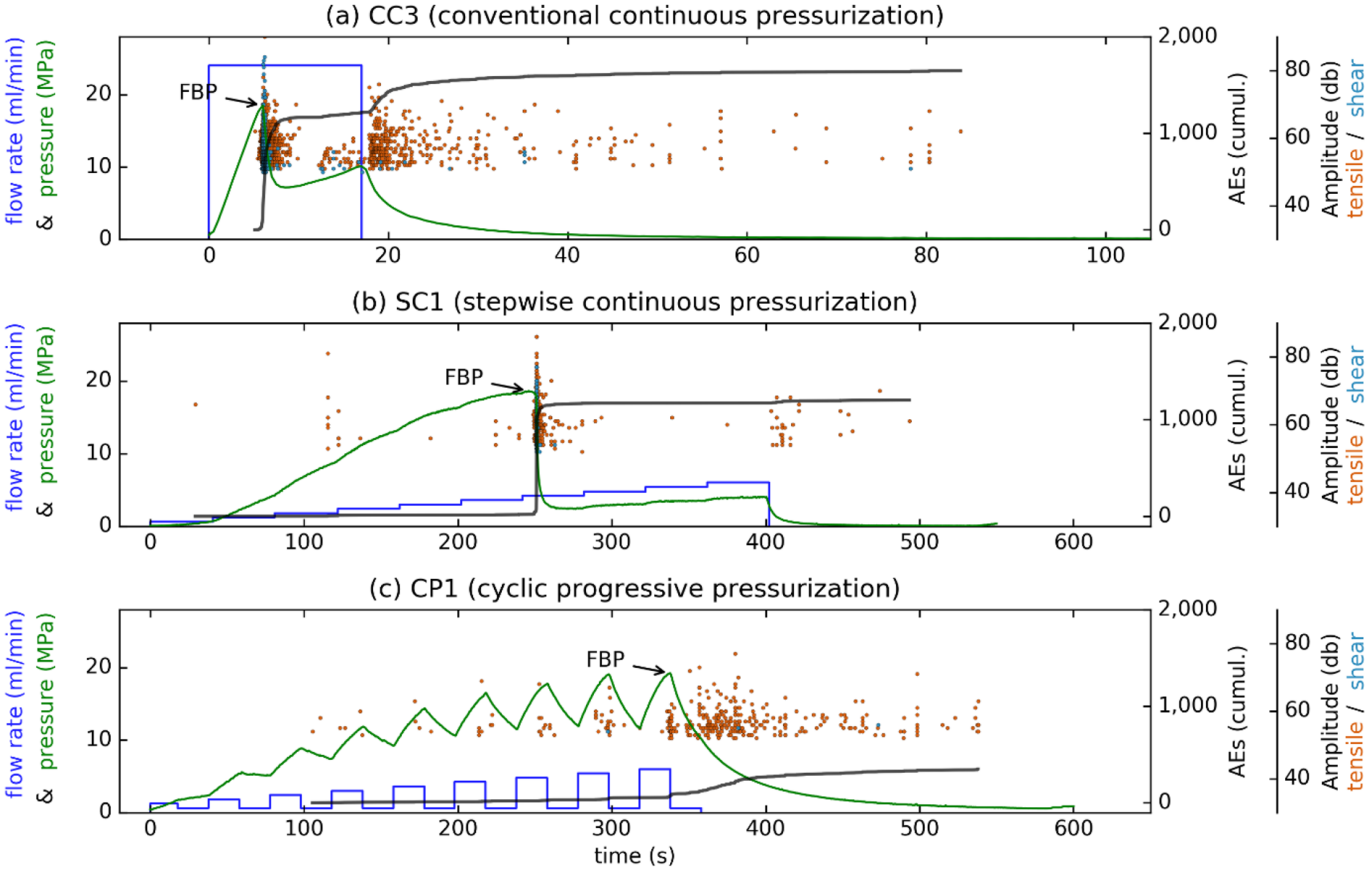
Laboratory hydraulic-fracturing results on true triaxially stressed Pocheon granite cubes with three fluid-injection schemes. Flow-rate-control tests with (**a**) conventional- continuous, (**b**) stepwise-continuous, and (**c**) cyclic-progressive pressurisation. *Red dots* indicate induced AE tensile failure; *light-blue dots* indicate induced AE shear failure. Other parameters were chosen to be analogous to those in Fig. 2.

The peak pressure during the first injection cycle after the integrity test corresponded to this FBP. As seen in Fig. 2, the breakdown pressure of fatigue test HF3 was 9.2 MPa, which was lower than the value obtained in the conventional tests in the same rock type (Fig. 3, HF1 and HF2, with FBP = 13.1 MPa and 10.9 MPa, respectively). The same tendency was observed in the neighbouring rock type, which was a fine-grained diorite gabbro^2^ (HF4 in Fig. S5a and HF5 in Fig. S5b, with FBP = 10.6 MPa and 9.0 MPa, respectively). No fatigue test could be performed in fine-grained granite due to the lack of fracture-free test intervals. The conventional test (Fig. S5c, HF6), however, was completed in this rock type. The full dataset of pressure-flow charts and seismic activity for these tests (HF4, HF5, and HF6) is provided in the supplement (Figure S5).

AE activity began to occur when the sealed section of the borehole was pressurised, except for with fatigue-hydraulic treatment (Fig. 2c, HF3 for times < 1300 s). AE activity decreased when the pressure in the interval was released and the borehole was shut in (Fig. 2, end of flow rectangle). Fracturing- and re-fracturing stages were accompanied by induced seismic events (Fig. 2, red dots). AE was absent in both post-shut-in- and cyclic-fatigue-fracturing stages only for AE events with magnitude estimation. Weaker events could also be found in the seismic catalogue in the post-shut-in and during cyclic injection^46^.

The total number of AEs in fatigue test HF3 was only half of the value of the conventional tests, although the detection threshold used and the amount of water injected were the same. In test HF3, the cumulative number of AEs was ~ 3000 as compared with ~ 6000 AEs for the conventional tests (Fig. 2, black curve). Moreover, the AE activity in the fatigue test began at a later stage in the treatment, and the maximum AE magnitudes observed tended to be lower compared with the conventional tests. The different total timings of the tests described in Fig. 2 resulted from the fact that impression-packer tests had been carried out at several stages of the hydraulic-fracturing operation. Fatiguing the rock has another striking side-effect: Post-shut-in seismic events seem to be suppressed when comparing conventional stimulation (Fig. 2a, b) with the hydraulic-fatigue test (Fig. 2c). One explanation for this finding may be the larger volume of rock that is affected by stress-release (relaxation) in fatigue injection compared with in conventional injection. If two or more fractures are generated at the wall of the stimulation interval, the pressure inside the fractures can communicate via the fluid volume. Instead of frequently observed post-shut-in seismic events in monotonic-injection tests (Fig. 2a, b), the fatigue operation may channelise the energy into rock fragmentation that lies below the seismic threshold because the energy released—as in “venting a valve” via monotonic-pressure increases—causes post-shut-in events with similar magnitudes compared with the pre-shut-in events, whereas the fatigue test allows for the release of smaller-magnitude events since the rock is gradually fragmented in fatigue cycles beforehand.

Figure 3 displays three hydraulic-fracturing tests on Pocheon granite cubes in the laboratory in which flow-rate-control injection was applied. Hydraulic parameters (Fig. 3, pressure, injection rate) are shown against time together with induced AE characteristics, such as amplitude and cumulative event number. In the conventional continuous test, the injection of 24 ml/min was stopped after breakdown had occurred, which was identified by a simultaneous decrease in pressure and an increase in AE activity (Fig. 3a, FBP = 18.5 MPa). The injection was stopped (shut in) ~ 11.5 s after breakdown had occurred. The pressure decreased, and the total number of AEs sharply increased at FBP (Fig. 3a, black curve), and then both continued to increase until shut-in had been reached. After shut-in, the total number of events did not increase considerably. The AE activity was highest at FBP, at which point most of the shear cracks formed (Fig. 3a, light blue dots). Tensile cracks occurred more continually (Fig. 3a, red dots).

In the stepwise progressive injection test (Fig. 3b), breakdown was identified by a clear drop in pressure during the seventh injection cycle (FBP = 18.6 MPa). Unlike in constant continuous injection, the accumulated number of AEs increased only slightly after shut-in. Unlike in the previous test, injection was not stopped after shut-in but continued as planned until it had reached a maximum-flow rate of 6 ml/min. As with continuous injection, the majority of AEs occurred at breakdown. In addition, it was at this point that most of the shear cracks developed (Fig. 3b), which indicated fracture propping.

In the cyclic-progressive injection test (Fig. 3c, fatigue test), the injection rate was increased gradually from 1.2 to 6 ml/min, but each stage also included phases of low injection (0.6 ml/min) that reduced the pressure within each cycle. For this case, no apparent breakdown pressure was observed, and the maximum recorded pressure was 19.2 MPa. Few AEs occurred before shut-in, which coincided with the high-pressure parts of each cycle. Although the AE activity increased after shut-in, the overall cumulative value was lower than for the other cases (Fig. 3, black curve). Almost no shear cracks occurred, which is in line with the apparent lack of breakdown pressure. The remaining diagrams of the pressure-controlled laboratory tests are presented in the supplementary material (Fig. S4).

### Fracture geometry- and permeability-enhancement process

**Figure 4:**
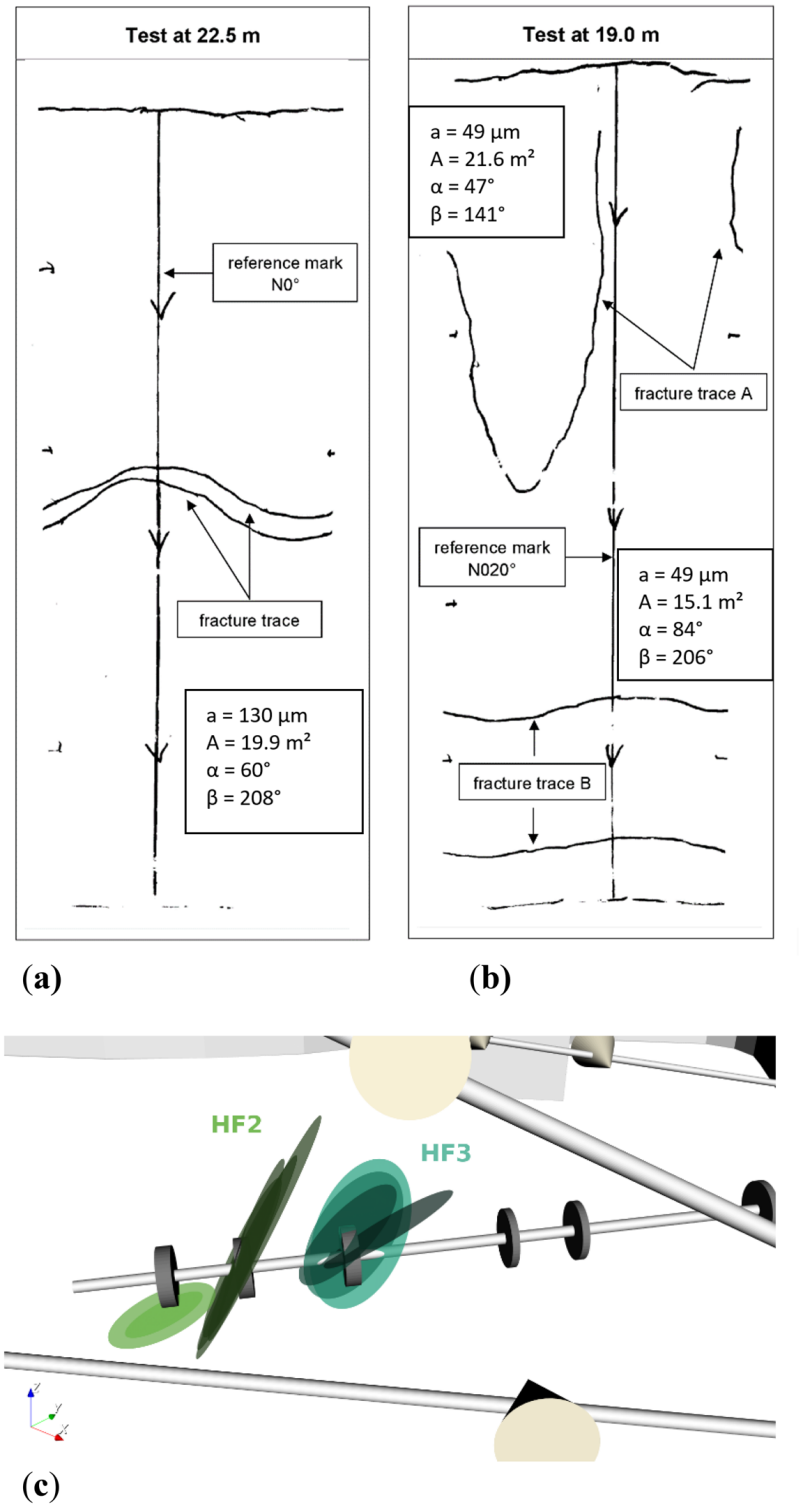
Integrated fracture data from impression packer with interval length of 0.75 m, back flow, and AE hypocentre analysis. Impression packer results shown are (**a**) from conventional test HF2 at a mid-interval depth of 22.5 m and (**b**) from fatigue test HF3 at a mid-interval depth of 19.0 m. Dip (α) and dip direction (β) of the fractures come from impression-packer analysis^48^. Fracture aperture (*a*) and area of fracture plane (*A*) come from AE -hypocentre extension and back-flow data^52^. In (**c**), fracture-plane orientations computed via full-waveform AE analysis are shown^46^.

**Figure 5:**
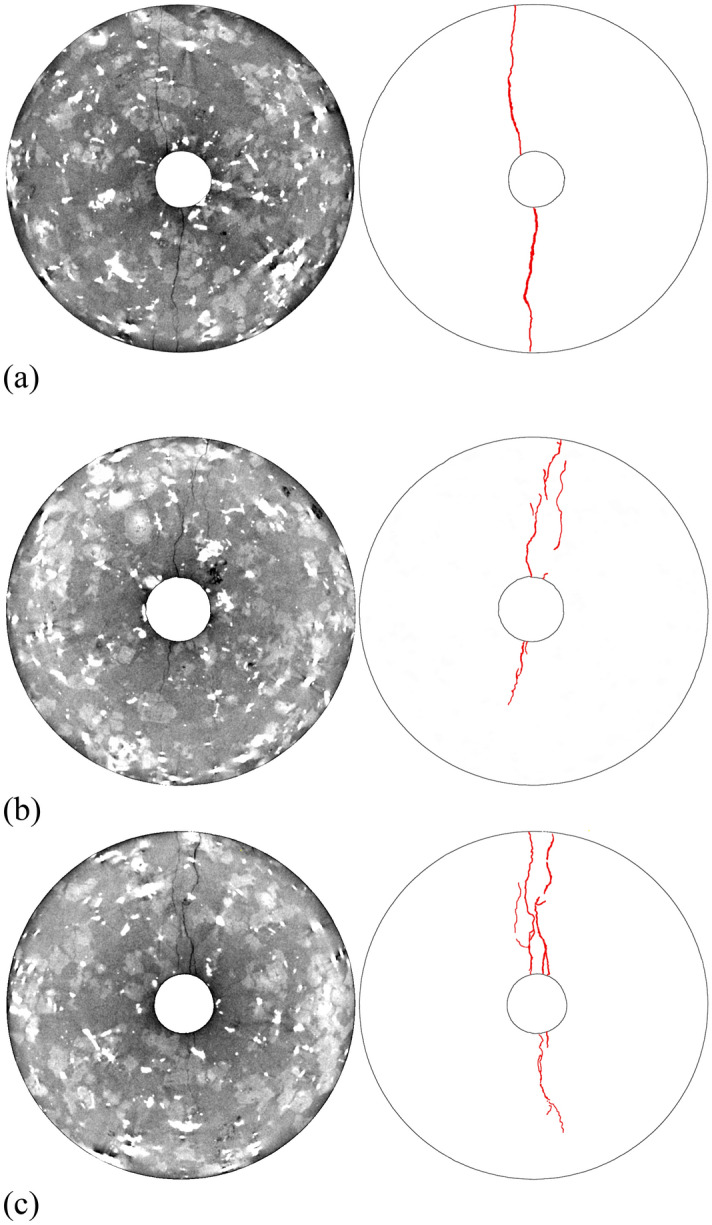
X-ray CT images (left) and fracture traces (right) after triaxial-injection tests on Pocheon granite cores (diameter: 50 mm; injection-borehole diameter: 8 mm). Continuous injection (**a**) led to single through-going bi-wing fractures, whereas cyclic injection with 43 cycles (**b**) and 150 cycles (**c**) led to multiple asymmetric fractures with a broader damage zone^49^.

In the following section, we discuss the fracture pattern in the granitic rock as revealed via impression-packer results in the mine (Fig. 4) and via X-ray CT-image analysis after laboratory hydraulic-fracturing experiments (Fig. 5). In Fig. 4, the resulting fracture patterns from two mine-injection tests that were conducted next to each other (Fig. 2, HF2 and HF3) are displayed as having been cause by the impression packer. While only one fracture trace could be found in the conventional test at a mid-injection interval depth of 22.5 m in the horizontal testing borehole (Fig. 4a, HF2), in the fatigue test, two fracture traces were visible at a mid-injection interval depth of 19 m (Fig. 4b, HF3 fracture trace A and B). Niemz et al.^46^ mapped the hydraulic fractures farther away from the wellbore using the expectation–maximisation algorithm. A single plane or multiples planes were fitted to the cloud of the AE hypocentres by maximising the expectation value of the underlying Gaussian distributions. During this process, outliers are assigned to a noise class, while the remaining hypocentres are attributed to a plane that is spanned by strike and rake. The estimated fracture planes support the observation of multiple fault planes with varying strike- and dip values for HF3. Our argument that the double-fracture phenomenon was caused by the fatigue-pumping scheme is as follows: Since the two branches of hydraulic fracture in HF3 developed directly from the borehole wall (as confirmed by impression-packer results and full-waveform AE-hypocentre tracking) and the wall of the test interval was found to be fracture-free before testing (BIPS borehole televiewer and core logs), we have strong support indicating that the double fracture resulted from the fatigue-testing scheme. We admit, however, that the in-situ stress deviator can play a significant role in fracture reorientation as soon as the fracture moves away from the injection interval. Moreover, pre-existing natural fractures in a rock mass can affect a growth path.

Figure 4c displays the results of hypocentre-expectation maximisation. The maximum extension of the fracture plane was assumed to correspond to the outer rim of the cloud of computed AE hypocentres. The average aperture of the hydraulic fractures was estimated using measured backflow values and fracture extension from maximisation expectation^52^. The conventional test generated a fracture of ~ 20 m^2^ with an aperture of ~ 130 µm. The fatigue test generated two fractures with a total fracture surface of ~ 37 m^2^ and an average aperture of ~ 49 µm each. This finding indicates that a different fracture- and permeability-evolution process occurred in hydraulic fatigue as compared with in conventional fracturing. The observation that the fracture geometry of HF3 is farther away from the injection interval, however, cannot have been caused by the pumping scheme alone. Other factors, such as pre-existing fractures or the stress-shadow effect of neighbouring fractures, could have played an equally important role.

However, in the laboratory-test results in which optical microscopy and X-ray CT images were used, the difference in fracture patterns caused by continuous and cyclic fluid injection into granitic rock was characterised quantitatively. Monotonic fluid injection caused single through-going bi-wing fractures to develop (Fig. 5a). Cyclic injection, on the other hand, led to multiple asymmetric fracture growths with greater tortuosity (Fig. 5b, c). Increasing the number of fluid-injection cycles led to more fractures in the process- and damage zone. Asymmetric fracture growth and the process-zone-enlargement mechanism are characteristic features of fatigue tests (Fig. 5b, c). Using optical microscopy, quartz fragments were found in the process zone of the fatigue fractures^50^. This finding strengthens our fatigue-hydraulic-fracturing concept, which postulated that rock chips would be generated by the secondary pump from open fracture walls (see supplementary material). For granitic rock, these natural proppants can be identified as the strongest minerals of the aggregate (here: quartz grains) (see Figure S7 in the supplement).

**Figure 6:**
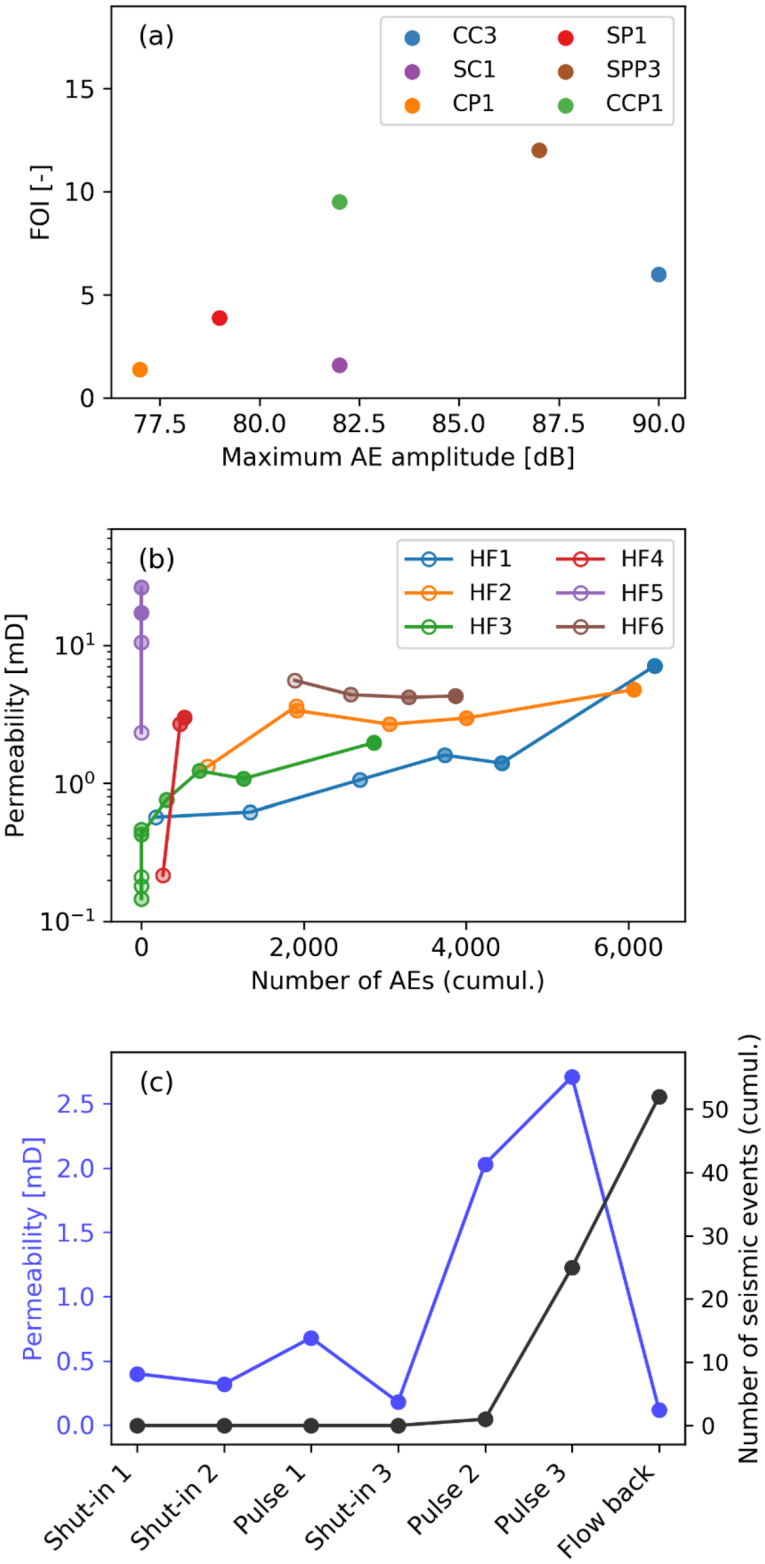
Permeability evolution across scale. (**a**) Fold of increase (FOI) of laboratory hydraulic fracturing test with different injection protocols: Flow-rate-controlled tests (CC3, SC1, CP1) and pressure-controlled tests (SP1, SPP3, CPP1). (**b**) Permeability enhancement for six field tests carried out in Äspö Hard Rock Laboratory: Conventional (HF1, HF2, HF4, HF6) and fatigue hydraulic tests (HF3, HF5). (**c**) Permeability development and seismicity observed during the field hydraulic test in the well PX1 in Pohang, South Korea.

The third part of the engineering puzzle involves the evolution of permeability. Below, we demonstrate that fatigue tests are able to enhance permeability, as documented in conventional hydraulic fracturing. Figure 6 summarises the permeability evolution across scales. At the laboratory scale (Fig. 6a), permeability enhancement is quantified as the fold of increase (FOI), which describes the ratio of injectivity after injection in comparison with the initial state before the treatment. This FOI is compared with the maximum magnitude of acoustic events via the different injection protocols. The greatest increase in the FOI is observed for the stepwise (SPP3) and cyclic (CPP1) pulsed experiments (see supplement, Figure S4). The conventional, constant-rate-injection test (CC3) reveals the greatest maximum magnitude of acoustic events.

In Fig. 6b, permeability enhancement versus the cumulative number of AEs is shown for the six field tests carried out at the Äspö Hard Rock Laboratory. The greatest permeability increase is observed for the pulsed hydraulic-fracture test, with progressively increasing flow rates and pulses on top (Fig. S5b, HF5) and without any seismicity observed. It should be noted that this test and HF4 were performed in fine-grained diorite gabbro. The cyclic hydrofrac test with a progressively increasing flow rate in Ävrö granodiorite (Fig. 2c, HF3) displayed the lowest observed seismicity when compared with the other conventional tests with constant-flow rates. The permeability evolution is among the highest, with intermediate absolute permeability occurring at the end of the test.

The permeability development and the seismicity observed during the field hydraulic test in the PX1 well in Pohang, Korea, is displayed in Fig. 6c. Permeability differs substantially for shut-in periods during cyclic injection with pulses and subsequent flow-back because the fracture system begins to open dynamically during the injection phases and begins to close subsequently in the shut-in phases and during the flow-back. Seismicity began at the very end of the treatment during pulsed injection due to the Kaiser effect^39^, which describes the delay of seismicity due to the previous stimulation in the well. Therefore, no comparison between conventional and fatigue-injection protocols is possible in terms of the occurrence of seismic events.

### Energy partition

**Figure 7:**
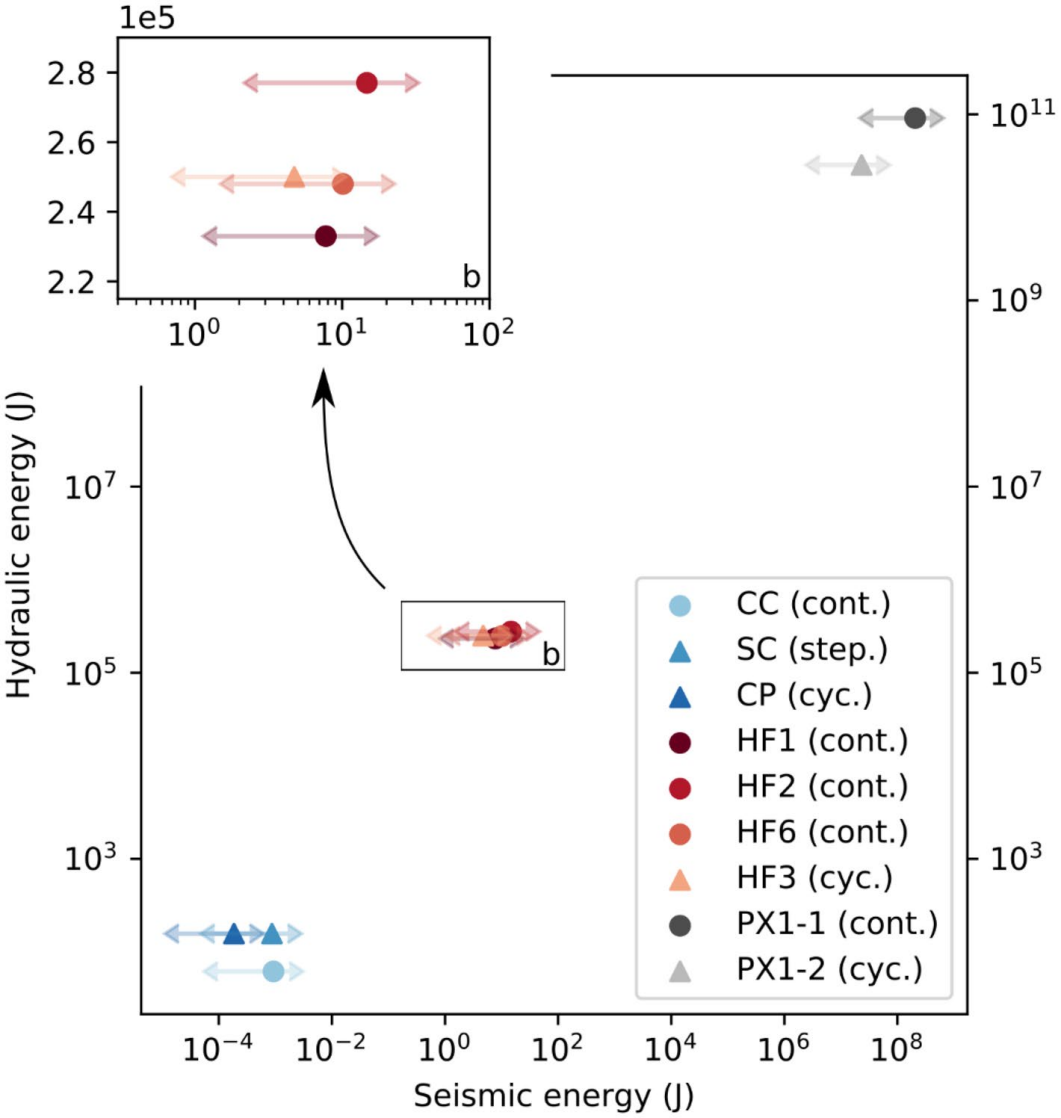
Hydraulic versus radiated seismic energy across scales.

**Figure 8:**
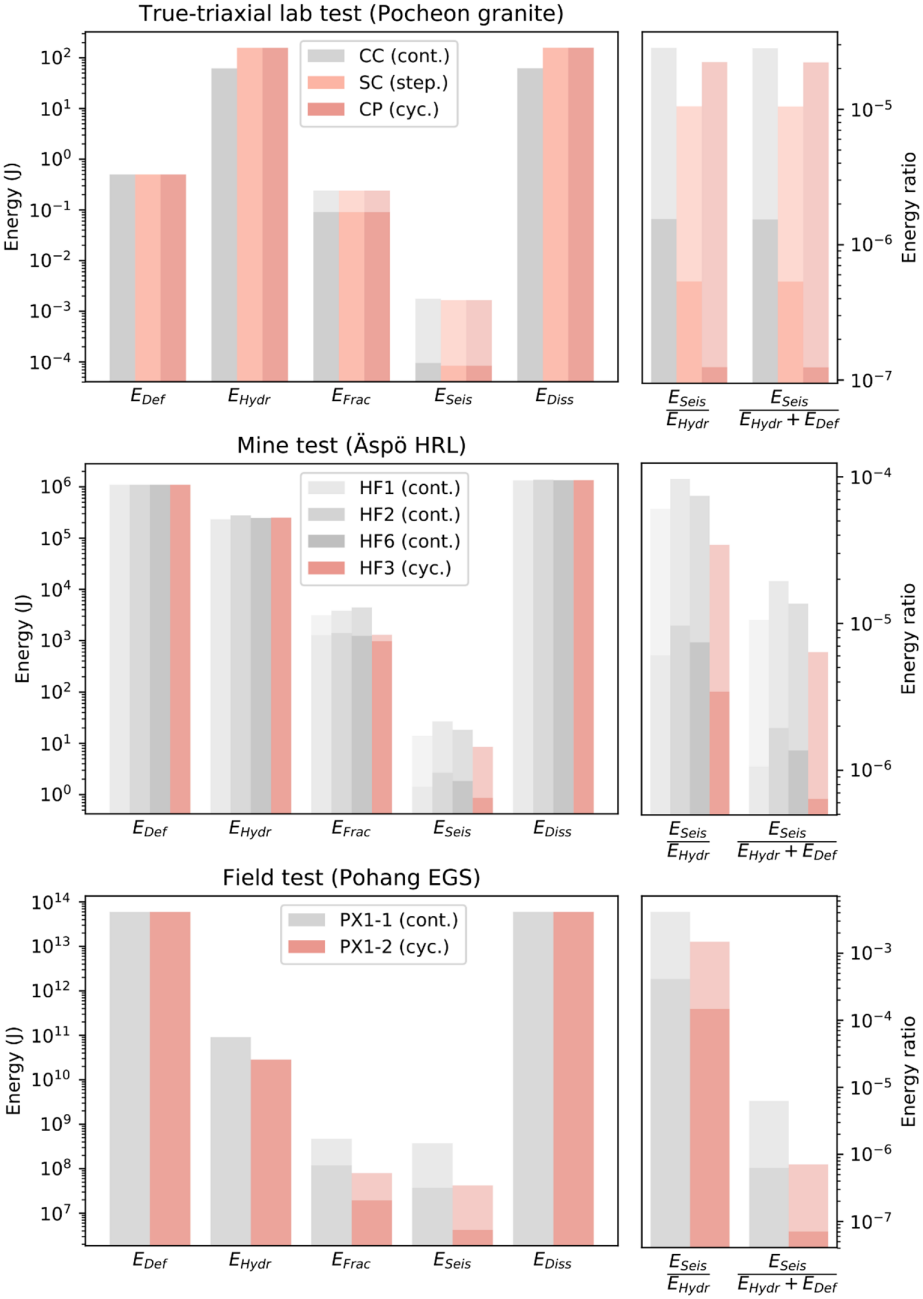
Energy partition in hydraulic-stimulation process. (**a**) True triaxial laboratory tests in Pocheon granite (CC = constant rate continuous, SC = stepwise rate continuous, CP = cyclic progressive); (**b**) underground test in naturally fractured granodiorite at Äspö HRL, Sweden (HF = hydraulic fracturing experiment); and (**c**) field stimulation at Pohang EGS site (PX1 = borehole PX1). All tests performed at all scales are flow-rate controlled.

In Figs. 7 and 8, energy values from laboratory-, mine-, and field-scale fluid-injection experiments are compared. In Fig. 7, the hydraulic energy is plotted against the radiated seismic energy throughout the three scales discussed in this study. Individual tests revealed the difference between conventional (continuous injection) and fatigue hydraulic fracturing (cyclic injection). The orders of difference between the hydraulic and seismic energy stem from the fact that the rock volume and fluid-injection volume were very different. The histograms in Fig. 8 reveal the complete set of energy values computed with the full dataset presented in the supplementary material (Table S8).

We observe a scale dependence in the ratio of radiated seismic and hydraulic energy, with values in the range of 1.9 × 10^–7^ to 6.7 × 10^–5^ for the laboratory tests (Fig. 8a), 3.4 × 10^–6^ to 9.7 × 10^–5^ for the mine tests (Fig. 8b), and 1.5 × 10^–4^ to 4 × 10^–3^ for the field tests (Fig. 8c). Regarding the injection styles, a trend of lower seismic-energy release can be seen for the cyclic-injection protocols. This finding highlights the efficiency of hydraulic-fatigue tests in flow-rate-control mode.

Energy ratios are displayed on the right of Fig. 8. In the laboratory tests, the values of *E*_*seis*_ with respect to *E*_*Hydro*_ (as well as *E*_*Hydro*_ and *E*_*Def*_) were not the same. The difference, however, was very small because *E*_*Def*_ in the small laboratory rock cube was small (0.5 J) when compared with *E*_*Hydro*_ (with values ranging from between 55 and 839 J). Therefore, the sum of both energy terms is dominated by the hydraulic part of the energy budget (see also Table S8). Both ratios E_seis_/E_hydr_ and E_seis_/(E_hydr_ + E_def_) were computed with the effective stress law and yielded similar values. This finding is independent of scale and within a range of two orders of magnitude. It appears that a larger rock volume that stored greater deformation energy also released more seismic energy.

The deformation energy computed from principal stresses and the rock volume was greater than the hydraulic energy in the mine- and field-scale injection tests. This result is mainly due to the rock volume involved in the injection tests. As uncertainty existed concerning the rock volume (especially for the field scale), the volume could have easily been overestimated by one order of magnitude. At the laboratory scale, the hydraulic energy was greater than the deformation energy. This partition of energy terms was directly related to the finite size of the rock samples. Finite rock volume is a general limitation of laboratory tests compared with mine- and field tests and is clearly documented in Fig. 8.

The fracture energy for tensile opening was greater than the radiated seismic energy in the laboratory-scale- and the mine-scale tests. In contrast, the field-scale values showed greater radiated seismic energy as an upper bound. This finding is likely related to the shearing of pre-existing fractures with relatively greater seismic energy release as compared with tensile fractures. Moreover, fracture energy in the field was estimated using the stimulated volume obtained via micro-seismicity observations only. In the Äspö HRL underground test, we had two independent sources of fracture-area estimates: one from the impression packer and one from the induced-seismic-cloud-extension- and back-flow measurements. In this regard, our underground tests were more reliable.

## Discussion of field application and evidence

The major findings in this study come from the joint interpretation of fluid-injection experiments at three scales with an underlying innovative mechanical concept of hydraulic-fatigue fractures. One category of findings (the engineering element) is related to the optimisation of the stimulation- and hydraulic-fracture-growth process, which is documented in breakdown pressure, fracture permeability, and induced-seismicity-evolution results. The second category of findings (the science element) is related to the energy budget of hydraulic fatigue versus the hydraulic-fracture process. In the following section, we discuss our new findings in relation to previous works.

Since the fatigue-hydraulic-fracturing concept was first introduced^37^ and cyclic and progressive pulse-injection schemes for hard rock at mine scale were first applied^2^, many authors have found evidence of differences between monotonic and cyclic fluid injection. In general, the benefit reported is threefold: First, hydraulic-fatigue testing allows the breakdown pressure to be lowered. Laboratory tests of cyclic hydraulic fracturing on Tennessee sandstone^53^, Pocheon granite^49^, Fontainebleau sandstone^54^, and cement-core plugs with variable strength properties^55^ have been reported. The percentage of the reported reduction of breakdown pressure varies for different rock types and with the porosity and strength of individual rock types. For Tennessee sandstone, the decrease in the breakdown pressure caused by cyclic fracturing can largely be attributed to the reduction in tensile strength due to water saturation^53^. In ultra-tight concrete, the reduction percentage is higher (25%) compared with in low- and medium-strength cement blocks (15%)^55^. This finding on concrete is in line with experiments on Xujiahe sandstone^56^. These authors reported smaller breakdown-pressure reduction (7%) in high-porosity samples (13%) as compared with a larger reduction (19%) in low-porosity sandstone (1%). In our study, we compared the reduction in breakdown pressure at the laboratory- and mine scales. In the supplement, we provide data on the cyclic-fatigue-breakdown pressure that have been normalised to the monotonic breakdown pressure of individual tests, and we plot this ratio against the log number of injection cycles (Figure S6). Data indicate a clear trend of breakdown-pressure reduction as a function of injection cycles. At the mine scale, we show data from two different rock types (Fig. S6, triangles). The 5-cycle HF3 experiment was performed in Ävrö granodiorite (Fig. S6, open triangle), while the 700-cycle HF5 experiment was performed in fine-grained diorite gabbro (Fig. S6, solid triangle). In fatigue test HF3, progressive cyclic injection was applied (Fig. 2), while in fatigue test HF5, a hydraulic hammer was used (i.e. cyclic pulse injection) (Fig. S5b). Therefore, the rock type and the fatigue-injection scheme in the mine can have an impact on breakdown-pressure reduction in Figure S6. However, more tests at the mine scale are required in order to separate individual impact factors on breakdown.

Second, fatigue-fluid-injection schemes have the power to modify the frequency-magnitude distributions of seismic events. Generally, the distribution can be described by the Gutenberg–Richter law^57^, in which the *b*-value represents the slope of the cumulative histogram of the observed magnitudes. An increase in the seismic *b*-value can be observed in all scales (see supplementary material). Although absolute *b*-values at different scales are difficult to compare due to magnitude-scaling issues, a general trend of relatively higher *b*-values for cyclic injections compared with for continuous injections could be observed at all scales (Fig. S3). This finding is new, and to our knowledge, has not been addressed in any previous work by other authors. An increase in *b*-values indicates a redistribution of seismic events towards smaller magnitudes. In the maximum-likelihood approach, the *b*-value is inversely related to the mean magnitude of the dataset (supplement, Eq. (13)). An increase in the *b*-value corresponds with a decrease in mean magnitude and thereby with more small-magnitude events or less large-magnitude events (or, in any case, with a larger number of smaller events compared with the number of large events) as long as the Gutenberg–Richter relation holds. Theoretically, the *b*-value itself is independent of the total number of events. However, small datasets with a limited number of events can have larger asymptotic errors in their *b*-value estimation. In the geothermal context, the mitigation of larger induced events is of utmost importance since many authorities rely on seismic traffic-light systems that include maximum-magnitude thresholds yet that sometimes neglect the total amount of released seismic energy. Many small events—even those with greater cumulative seismic energy than might be expected for *b*-values larger than 1.5—would not cause a red light and halt the injection activity. If designed properly, cyclic injection can systematically replace several larger-magnitude seismic events with a larger number of smaller-magnitude seismic events because hydraulic fatigue is an efficient rock-fragmentation process (see supplement, fracture-mechanics formulation of hydraulic fatigue). Future tests should search for injection parameters that minimise the seismic-energy release. We suggest evaluating the effect of fracturing-fluid viscosity in combination with—inter alia—the number of injection cycles, crack resting times, duration times, amplitudes, and the phase shift of pressurisation intervals. Although the total energy budget in situ is fixed, with some limits, the partition of seismic and fracture-surface energy during the rock-degradation process is optimisable.

Fracturing fluids are known to exert an impact on the hydraulic-fracture-growth process. A variety of fluids are commonly used in the laboratory, including freshwater, oil, CO_2_ (liquid, super-critical), and gas (CO_2_, N_2_). The viscosities of these fluids can range from between several orders of magnitude (10^–2^ and 10^–6^ mPa s). Results indicate that the viscosity of the injection fluid exerts a significant impact on the hydraulic fracturing of granites^58,59^ as well as of other rock types^60,61^. Ishida et al.^62^ compared four different fluids of super-critical and liquid CO_2_, water, and viscous oil with a low to high viscosity of 0.051 to 337 mPa s and confirmed that breakdown pressure increases with increasing viscosity.

The impact of fluid viscosity on hydraulic-fracture growth is seen as follows: For a given rock type, high-viscosity fluids have a smaller infiltration rate compared with low-viscosity fluids, even at the same injection rate. As a result, the rock can be fractured at different breakdown pressures. Jung et al.^63^ compared the total volume of injection fluid infiltrated into granite samples when using water and various oil-based fluids (80, 122, and 152 mPa s) at the same constant-injection rate. The measured results reveal that the total amount of oil infiltration is about half that of water infiltration. Breakdown pressure by oil fracturing is about two times that by water fracturing, which is explained by a shift in fracturing behaviour from viscosity-dominated- to toughness-dominated regimes^64^.

Changing the fracturing fluid in hydraulic fatigue can be beneficial in designing short and compact- versus long and persistent fractures. If short and compact fractures are desired, water-hammer fracturing can be applied, while if long and persistent fractures are desired, highly viscous gel and cyclic-progressive fatigue tests can be applied. The hydraulic-fatigue concept can also be of value for field EGS applications when massive conventionally stimulated cloud-like fractures need to be replaced by controlled multi-stage fractures for the sake of optimising geothermal heat exchangers.

Third, compared with monotonic injections, cyclic fluid injections into the geo-reservoir have been demonstrated to increase the hydraulic performance of the fracture network^54^. This finding has important implications for EGS stimulations, in which an increase in reservoir permeability and dilatancy would enhance reservoir productivity. Noel et al.^54^ demonstrated that a dilatancy threshold exists (~ 1% for Fontainebleau sandstone) after which macroscopic failure occurs. For reservoir applications, approaching this critical dilatancy could provoke fast failure of the reservoir and associated induced seismic activity. We recommend applying hydraulic fatigue in order to better control fracture growth and induced seismicity at a level below the critical dilatancy threshold, where other stimulation methods may fail. Controlling fracture growth via hydraulic fatigue, on the other hand, goes hand in hand with sophisticated stimulation techniques and longer treatment times.

In mine testing, a combination of cyclic-progressive and pulse-hydraulic fracturing yields the best increase in permeability (Fig. 6b). To our knowledge, combining cyclic- and pulse-pumping schemes is a new concept and has not been written about by other authors. Cyclic injection promotes the development of more fractures in a broader zone as has been documented in laboratory tests on sandstone and granite. The extension of the fracture process zone in Tennessee sandstone after cyclic hydraulic fracturing has been reported to be about twice that of the process zone in the conventional treatment^53^. The corresponding increase in fracture permeability by cyclic injection into Tennessee sandstone has been reported to correspond to a factor 3–10 times higher than that of conventional hydraulic fracturing. Zhuang et al.^50^ reported a denser network of grain-boundary shear cracks in Pocheon granite after hydraulic-fatigue testing as compared with conventional treatment with primarily intra- and inter-granular tensile cracks in a narrow band. In the same study, quartz grain fragments in the main fracture were reported as being natural proppants after hydraulic-fatigue testing.

Simple cyclic pumping has also been used in shale-gas fracturing and has been demonstrated at the field scale with the concept of “relax a frac”, in which part of the stimulation treatment is pumped, followed by an extended shutdown to relax the formation^65^. In addition, perforation clusters have been demonstrated at the laboratory scale to be able to efficiently stimulate multiple fractures in horizontal wells^66^. By applying fatigue hydraulic fracturing with the reported gain in permeability enhancement (one order of magnitude in mine testing)^48^, perforated facture stages can superimpose individual permeability performance, which renders the treatment more efficient for shale-gas production. Perforation clusters can also be used in geothermal (EGS) development.

First attempts have also been made to apply cyclic stimulations in the field of EGS. In the cyclic soft-stimulation-concept treatment performed in August 2017 at the Pohang EGS site, a total of 1756 m^3^ of surface water was injected into the PX-1 well at flow rates of between 1 and 10 l/s, with a maximum wellhead pressure of 23 MPa^39^. During the treatment, a total of 52 induced micro-earthquakes were detected in near-real-time. The largest event had a magnitude of Mw = 1.9, which was below the critical-threshold level of Mw = 2.0 set in advance. A second project using varying flow-rate stimulation and a near-real-time seismic-event-control concept in the framework of EGS stimulation was performed one year later at the campus of Aalto University, located at Espoo near Helsinki, Finland. In June and July 2018, a total of 18,160 m^3^ of fresh water was pumped into crystalline rocks at a depth of 6.1 km over 49 days^67^. The locations, magnitude, and evolution of seismic and hydraulic energy were used to control hydraulic-fracture growth and stabilisation during the stimulation treatment in line with the fatigue-hydraulic-fracturing concept.

The focus of this study was on optimising fluid injection, induced seismicity, and permeability evolution during the hydraulic-fracture-growth process in naturally fractured granitic rock masses. At this point, it is necessary to indicate that fluid injection close to or directly into a fault is a different problem, as is documented, for example, by the detection of “runaway fractures” via fluid-injection-induced seismicity at EGS sites^21,25,68^.

Relaxation damage control via hydraulic-fatigue cycles not only seems to work in granite at the grain-boundary scale (laboratory) and at the scale of naturally fractured granitic rock mass (mine), but it is also a candidate for being applied at the field scale. The basic ingredients in cyclic fatigue testing—including variable flow rates, multiple crack-tip resting times, and more-tortuous and denser fracture-network evolutions—can be seen scale-independently, although the intrinsic properties of different rocks are involved at various scales.

We admit that more rock types need to be investigated in the future, and each individual rock type may need a tailor-made cyclic- and pulse-injection scheme in order to increase the overall confidence in the hydraulic-fatigue concept presented in this study.

## Methods: computation of radiated seismic-, fracture-surface- and hydraulic energy

We compared hydraulic-fracture nucleation- and growth process by analysing seismic signals recorded with high-frequency acoustic-emission (AE) sensors, including their magnitude–frequency distribution, radiated seismic energy, and cumulative seismic-energy release. Second, we analysed hydraulic parameters in terms of formation-breakdown pressure and permeability evolution. Third, we estimated fracture geometrical parameters—such as aperture, area, and tortuosity—using AE hypocentre locations in the mine test as well as micro-X-ray CT image analysis of granite specimens after the laboratory test.

We estimated radiated seismic energy (*E*_*Seis*_) via an AE analysis. We computed fracture-surface energy (*E*_*Frac*_) via fracture-geometry data and experimentally determined fracture-toughness data. We computed hydraulic energy (*E*_*Hydr*_) via pressure–time charts and the net volume of fluid injected. We did not know the value of energy dissipated during the hydraulic-fracturing process (*E*_*Diss*_). Using a rough estimate of the stored elastic strain energy of the granite cubes stimulated under stress at different scales (*E*_*Def*_), we computed a lower-bound value (*E*_*Diss*_). The energy balance of a change in a given stress state (i.e. hydraulic stimulation) was given by Eq. (2):

2 $${E}_{Def}+{E}_{Hydr}={E}_{Seis}+{E}_{Frac}+{E}_{Diss}.$$

For more details about computing and estimating individual energy terms, please consult the supplementary material section.

## Supplementary Information

Supplementary Information

## Data Availability

The datasets generated and/or analysed during the current study are summarised in Supplementary Table S8. Raw and unprocessed data are available upon request at niemz@gfz-potsdam.de.
