# Supplementary material for: Relaxation damage control via fatigue-hydraulic fracturing in granitic rock as inferred from laboratory-, mine-, and field-scale experiments
Source: Sci Rep. 2021 Mar 24;11:6780. doi: 10.1038/s41598-021-86094-5 (PMC7991663; doi:10.1038/s41598-021-86094-5)
Supplement: Supplementary file 1 — Supplementary Information [file 41598_2021_86094_MOESM1_ESM.docx]

**Supplementary material for the article:**

**Relaxation damage control via fatigue-hydraulic fracturing in granitic rock as inferred from laboratory-, mine- and field-scale experiments**

Arno Zang^1,2*^, Günter Zimmermann^1^, Hannes Hofmann^1^, Peter Niemz^1,2^, Kwang Yeom Kim^3^, Melvin Diaz^3^, Li Zhuang^4^, Jeoung Seok Yoon^5^

^1^German Research Centre for Geosciences GFZ, Telegrafenberg, 14473 Potsdam, Germany

^2^Institute of Geosciences, University of Potsdam, 14469 Potsdam, Germany

^3^Korea Maritime and Ocean University, Busan, Republic of Korea

^4^Korea Institute of Civil Engineering and Building Technology, Goyang, Republic of Korea

^5^DynaFrax UG (limited), Helmholtzstr. 6, 14467 Potsdam, Germany

^*^Corresponding author: Arno Zang (zang@gfz-potsdam.de; +49 331 2881325); GFZ Section 2.6 Seismic Hazard and Risk Dynamics

**This file contains:**

- Supplementary methods: fracture-mechanics formulation of hydraulic fatigue
- Energy partition of hydraulic fracturing in granitic rock across scales
- Analysis of seismic *b*-value, additional fluid-injection experiments, normalised breakdown pressure and microscopic inspection of rock chips
- Supplementary Figures S1 to S7
- Supplementary Table S8

**Supplementary methods**

**Fracture-mechanics formulation of hydraulic fatigue**

The characteristic difference between conventional hydraulic fracturing with continuous pumping and a single flow rate on the one hand and fatigue hydraulic fracturing engineered by cyclic and pulse pressurisation with multiple flow rates on the other hand lies in the formation of the fracture-process zone. A synoptic picture of the process zone in hydraulic fatigue as compared with monotonic injection in conventional hydraulic fracturing is provided by Zang et al.^1^ (see Figure S1). The key component of the process-zone development in hydraulic fatigue is the relaxation of crack-tip stresses. Relaxation damage is controlled (a) by frequent phases of pressurisation and depressurisation, for example, by cyclic fluid injection, and (b) by pulse-pressure oscillations that are superimposed using a second pump^2^. In contrast to the Kiel process^3^, secondary-pump oscillations efficiently generate rock chips from the fracture walls (natural proppants). In line with the Kiel process, the proppants are transported to the crack tip and change the stress field locally. The fatigue-fracture process zone is enlarged by multiple branching fractures (Fig. S1, FHF). Its size and individual micro-fracturing sources are different from classical fracture mechanics (Fig. S1, HF).

Rinne et al.^4^ created a fracture-mechanics representation of mechanical fatigue (their Figs. 2.8 and 2.9). In Figure S2, we schematically modified these figures for the case of hydraulic fatigue. First, the sinusoidal excitation in mechanical fatigue (Fig. S2a, *black curve*) shows relatively higher values of the stress-intensity factor *K* compared with the sinusoidal excitation in hydraulic fatigue (Fig. S2a, *blue curve*) because *K* depends on the degree of saturation. Moreover, the difference between the maximum and minimum stress-intensity factor *ΔK* is different in both scenarios. Second, in Figure S2b, the increment in fracture length per loading cycle (*da/dN*) is displayed in a double-logarithmic plot, where *ΔK_th_* is the stress-intensity-factor difference below which no crack growth occurs. Critical crack growth in fatigue begins at a threshold value referred to as the critical stress-intensity-factor difference, or *ΔK_IC_*. In Figure S2b, three phases of crack propagation are distinguished: I = decelaration, II = stationary, and III = acceleration. Crack growth can be described by the *Donahne law* in Phase I, by the *Paris-Erdogan law* in Phase II, and by the *Forman law* in Phase III^4^. Unified Eq. (1) – the so-called *Erdogan-Ratwani law* – covers all three phases of crack propagation in mechanical-fatigue tests with three material constants: *c*, *m*, and *n*.

$\frac{da}{dN}=\frac{C{(1+\beta)}^{m} \cdot{(\Delta K - \Delta K_{th})}^{n}}{K_{C}-\left( 1+ \beta\right) \cdot\Delta K}$; $\beta= \frac{K_{max}+ K_{min}}{K_{max}- K_{min}}$ (1)

This concept of mechanical fatigue can be translated into the new concept of hydraulic fatigue. In so doing, stress-intensity factors from dry rock are replaced by stress-intensity factors of saturated rock. Second, mechanical loading cycles (*N*) are replaced by pressure cycles (*Np*) in the *Erdogan-Ratwani law*. Eq. (2) respresents the hydraulic-fatigue equivalent to eq. (1) and is introduced as *Erdogan-Ratwani-Zang* law.

$\frac{da}{dNp}=\frac{C{(1+\beta)}^{m} \cdot{(\Delta K_{wet} - \Delta K_{th,wet})}^{n}}{K_{C, wet}-\left( 1+ \beta\right) \cdot\Delta K_{wet}}$ (2)

**Energy partition of hydraulic fracturing in granitic rock across scales**

*Hypothesis*. In the conventional hydraulic-fracturing test, the injection pressure is increased monotonically until fracture breakdown occurs (single flow-rate test). As a consequence, the hydraulic energy pumped into the rock mass is an end-member maximum value that is much larger than the dissipated fracture- and friction energy, the plastic energy, and the radiated seismic energy. In hydraulic fatigue (variable flow rates), the crack-tip stresses are relaxed multiple times, and a different energy balance follows that is characterised by a total hydraulic energy input that is comparable to the dissipated energy from fracturing-, friction-, plastic-, and radiated seismic energy. As a result, variable flow-rate tests allow multiple branching fractures to be formed ahead of the fatigue-fracture tip and leave behind a broader damage-zone fracture network that also makes a difference in permeability enhancement as compared with conventional fracturing. Hydraulic fatigue is an optimised energy-conversion process. A significant portion of the hydraulic energy is converted into damage and fracturing of the rock mass using pressure cycles and pulses. Since energy dissipation and damage evolution are both stress-path-dependent, so too is fatigue hydraulic fracturing.

*Energy computation*. To quantify the energy-partition process, we isolated deformation energy and hydraulic energy on the input side of the energy equation and isolated the fracture energy, radiated seismic energy, and dissipated energy on the consumption side. Table S8 lists all parameters introduced in the following sections as well as energy estimates in all three scales.

The following assumptions and equations were used to compute individual energy terms.

*Deformation (strain) energy*

The deformation (strain) energy (*E_Def_*) within a finite rock volume (*V*) that was subjected to three effective principal stresses ($\sigma_{1}^{'}>\sigma_{2}^{'}>\sigma_{3}^{'}$; principal stress minus formation pressure, here assumed to be hydrostatic) was calculated by

$E_{def}=\sum_{i=\sigma_{1}^{'}}^{\sigma_{3}^{'}} \frac{V}{2E}i^{2}$, (3)

with *E* being the rock Young’s modulus. At the laboratory scale, the volume corresponded to the sample size. At the mine- and field scales, the rock volume was chosen based on the scatter of AE and seismic sources, thereby providing a rough estimate of the activated/stimulated volume.

*Fracture (surface) energy*

The energy required to open a pure tensile fracture (*Mode I*) in intact rock (*E_Frac_*) was calculated using the total rupture (fracture) area (*A_r_*) and the energy-release rate (*G_IC_*):

$E_{Frac}=G_{IC}A_{r}$, (4)

with

$G_{IC}=\frac{(1-\upsilon^{2})K_{IC}^{2}}{E},$ (5)

where *K_IC_* is the stress-intensity factor for *Mode-I* fractures and *υ* is the rock Poisson’s ratio. Eq. (5) is valid for plane-strain conditions. To account for variabilities in *K_IC_*, we used upper- and lower-bound values of 1.05 and 1.58 MPa m^1/2^, respectively, from minimum and maximum mean values determined via three-point bending tests on Pocheon granite^5^. Values of *K_IC_* were used for energy computations throughout all scales. Ambient *K_IC_* values were corrected for confining stress conditions^6^:

$K_{IC\_corr}=K_{IC\left( 0.1MPa \right)}\left( 1+0.037\sigma_{3}^{'} \right).$ (6)

The corrected value of the stress-intensity factor was used in Eq. (5) to calculate the energy-release rate (*G_IC_*). We estimated the fracture area (*A_r_lab_*) using the laboratory tests of Zhuang et al.^4^. In so doing, we used the hydraulic-fracture half-length (*L_f_*) and the length (*h*) of the open-hole section of the injection borehole (70 mm) as the fracture-height upper bound:

$A_{r\_lab}=2L_{f}h$. (7)

In the mine-scale experiment, the fracture-surface area was inferred from the extension of the acoustic-emission cloud^7^. In the field scale, we used the cumulative fracture area of all seismic events from Hofmann et al.^8^, which was based on the empirical scaling relation between moment magnitude (*Mw*) and the rupture area from Wells and Coppersmith^9^:

$A_{r\_field}={10}^{\frac{Mw-3.49\pm0.16}{0.91\pm0.03}}.$ (8)

As for *K_IC_*, we set upper- and lower-bound values for the fracture areas (Table S8).

*Hydraulic energy*

The hydraulic energy (*E_Hydr_*) resulting from an injection during time *t* with downhole pressure *P* and volume-flow rate $\dot{V}$ was calculated according to Goodfellow et al.^10^:

$E_{Hydr}=\int_{0}^{t} P\dot{V}dt.$ (9)

*Radiated seismic energy*

Following Hanks and Kanamori^11^, the radiated seismic energy *E_Seis_* was calculated by

$E_{Seis}=\frac{{\Delta\sigma m}_{0}}{2\mu}$, (10)

with stress drop *Δσ*, shear modulus *μ*, and scalar seismic moment *m_0_*, assuming earthquake self-similarity. Kwiatek et al.^12^ demonstrated that this assumption is reasonable for the Äspö experiment. Stress drop is commonly assumed to be between 1 and 10 MPa^13^, but 0.1 MPa has also been reported^14^. In this study, we estimated *E_Seis_* with a lower-bound stress drop of 0.1 MPa and an upper-bound stress drop of 1 MPa. The rock’s shear modulus (*μ*) was calculated using Young’s modulus and the Poisson ratio (Table S8). Using the definition of moment magnitude (*M_w_*) for *m_0_* in Nm^13^, we calculated the scalar seismic moment:

$m_{0}={10}^{1.5M_{w}+9.1}.$ (11)

For Äspö mine-scale experiments, we applied a magnitude scaling that assumed a linear relationship between the relative magnitude (*M_AE_*) used in the extended-event catalogue^7^ and *M_w_* as calculated for a subset of larger events^12^. A best-fitting linear regression for *M_w_*- and *M_AE_* estimates of 195 events contained in both catalogues was calculated using the RANSAC algorithm^15^.

At the laboratory-test scale, we calibrated the AE sensors via ball-drop experiments. Small steel spheres (diameter: 1 mm and 2 mm) were dropped onto a granite block from a pre-defined height of 100–200 mm with same AE-sensor setup and sensitivity as was used in the true triaxial experiments. In this manner, we were able to directly relate the AE amplitude provided by the AE recording- and analysis system (MITRAS) to the potential energy resulting from the impact of the sphere. Subsequently, we applied the log-amplitude–log-energy relation from the ball-drop calibration experiment to the event amplitudes from the true triaxial-deformation experiments.

*Dissipated energy*

The dissipated energy (*E_Diss_*) was calculated via Eq. (2) in the main text:

$E_{Diss}=E_{Hydr}+E_{Def}-E_{Frac}-E_{Seis}$. (12)

**Analysis of seismic *b*-value, additional fluid-injection experiments, normalised breakdown pressure and microscopic inspection of rock chips**

*Hypothesis*. The mechanism leading to a reduction in event magnitude by cyclic injection was hypothesised to be the development of a larger number of smaller events compared with fewer large events caused by continuous injection. One reason for this development was the division of the injected hydraulic energy into smaller parts, which was expected to lead to a division of the radiated seismic energy.

*Computation of b-values*. For all scales, we consistently estimated the Gutenberg–Richter *b*-values^16^ using a maximum-likelihood approach corrected for measurement errors and magnitude binning^17,18^:

$b=\frac{1}{\ln\left( 10 \right)\Delta M}\ln\left( p \right)$, (13)

with

$p=1+\frac{\Delta M}{\hat{\mu}-M_{c}}$, (14)

magnitude of completeness $M_{c}$, bin size$\Delta M$ and mean magnitude $\hat{\mu}$. $M_{c}$ was determined independently by applying a bootstrap-based change-point detection method^19^.

**References**

1. Zang, A. *et al.* How to reduce fluid-Injection-induced seismicity. *Rock Mech. Rock Eng.* **52**, 475–493, DOI: 10.1007/ s00603-018-1467-4 (2019).
2. Zang, A. *et al.* Hydraulic fracture monitoring in hard rock at 410 m depth with an advanced fluid-injection protocol and extensive sensor array. *Geophys. J. Int.* **208**, 790–813, DOI: 10.1093/gji/ggw430 (2017).
3. Kiel, O. M. The Kiel process - reservoir stimulation by dendritic fracturing. Tech. Rep., Society of Petroleum Engineers (1977).
4. Rinne, M., Stephansson, O., Shen, B. & Konietzky, H. Introduction to the theories of rock fracturing. In *Modelling Rock Fracturing Processes*, 7–24 (Springer, 2020).
5. Zhuang, L. *et al.* Laboratory true triaxial hydraulic fracturing of granite under six fluid injection schemes and grain-scale fracture observations. *Rock Mech. Rock Eng.* DOI: 10.1007/s00603-020-02170-8 (2020).
6. Müller, W. Brittle crack growth in rocks. *Pure and Applied Geophysics* **124**, 693–709, DOI: 10.1007/BF00879605 (1986).
7. Niemz, P. *et al.* Full-waveform-based characterization of acoustic emission activity in a mine-scale experiment: A comparison of conventional and advanced hydraulic fracturing schemes. *Geophys. J. Int.* **222**, 189–206, DOI: 10.1093/gji/ ggaa127 (2020).
8. Hofmann, H. *et al.* First field application of cyclic soft stimulation at the Pohang Enhanced Geothermal System site in Korea. *Geophys. J. Int.* **217**, 926–949, DOI: 10.1093/gji/ggz058 (2019).
9. Wells, D. L. & Coppersmith, K. J. New empirical relationships among magnitude, rupture length, rupture width, rupture area, and surface displacement. *Bull. Seismol. Soc. Am.* **84**, 974–1002 (1994).
10. Goodfellow, S. D., Nasseri, M. H. B., Maxwell, S. C. & Young, R. P. Hydraulic fracture energy budget: Insights from the laboratory. *Geophys. Res. Lett.* **42**, 3179–3187, DOI: 10.1002/2015GL063093@10.1002/(ISSN)1944-8007.FRACKING (2016).
11. Hanks, T. C. & Kanamori, H. A moment magnitude scale. *J. Geophys. Res. Solid Earth* **84**, 2348–2350, DOI: 10.1029/ JB084iB05p02348 (1979).
12. Kwiatek, G. *et al.* Insights into complex subdecimeter fracturing processes occurring during a water injection experiment at depth in Äspö Hard Rock Laboratory, Sweden. *J. Geophys. Res. Solid Earth* **123**, 6616–6635, DOI: 10.1029/2017JB014715 (2018).
13. Kanamori, H. & Brodsky, E. E. The physics of earthquakes. *Reports on Prog. Phys.* **67**, 1429–1496, DOI: 10.1088/ 0034-4885/67/8/R03 (2004).
14. Collins, D. S. & Young, R. P. Lithological controls on seismicity in granitic rocks. *Bull. Seismol. Soc. Am.* **90**, 709–723, DOI: 10.1785/0119990142 (2000).
15. Fischler, M. A. & Bolles, R. C. Random sample consensus: A paradigm for model fitting with applications to image analysis and automated cartography. *Commun. ACM* **24**, 381–395, DOI: 10.1145/358669.358692 (1981).
16. Gutenberg, B. & Richter, C. F. Frequency of earthquakes in California. *Bull. Seismol. Soc. Am.* **34**, 185–188 (1944).
17. Tinti, S. & Mulargia, F. Confidence intervals of b values for grouped magnitudes. *Bull. Seismol. Soc. Am.* **77**, 2125–2134 (1987).
18. Marzocchi, W. & Sandri, L. A review and new insights on the estimation of the b-value and its uncertainty. *Annals Geophys.* **46**, 1271–1282, DOI: 10.4401/ag-3472 (2009).
19. Amorèse, D. Applying a change-point detection method on frequency-magnitude distributions. *Bull. Seismol. Soc. Am.* **97**, 1742–1749, DOI: 10.1785/0120060181 (2007).
20. Stille, H. & Olsson, P. Summary of rock mechanical results from the construction of Äspö Hard Rock Laboratory. *Stock. SKB Prog. Rep. HRL-96-07* (1996).
21. Kwon, S. *et al.* Characterization of 4.2-km-deep fractured granodiorite cores from Pohang geothermal reservoir, Korea. *Rock Mech. Rock Eng.* **52**, 771–782, DOI: 10.1007/s00603-018-1639-2 (2019).

**Supplementary Figures**

**Figure S1**. The process zone in hydraulic fatigue.


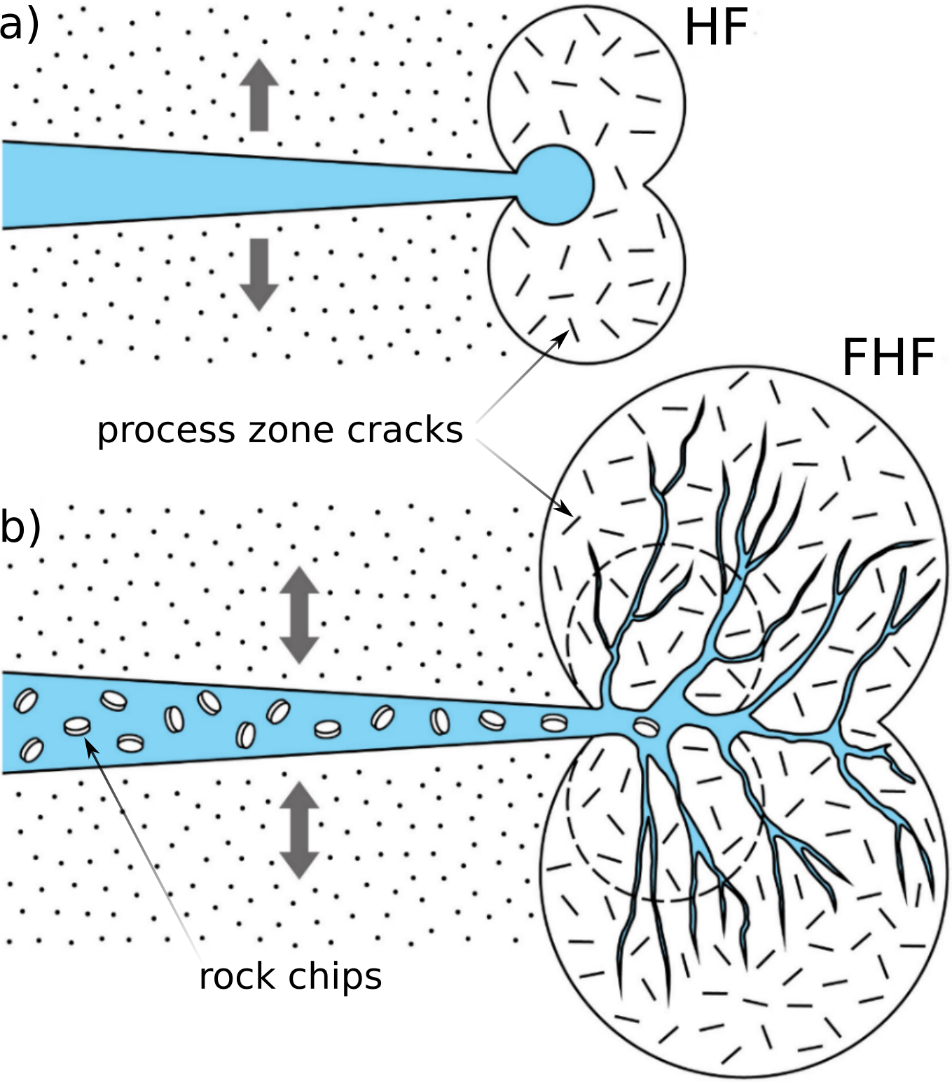


**Figure S1**. Fracture-mechanics approach to hydraulic fatigue. Synoptic picture of a fracture process zone developing in (**a**) conventional hydraulic fracturing with continuous fluid injection and a kidney-shaped Mode-I secondary-cracking area at the crack tip and (**b**) fatigue hydraulic fracturing with progressive and dynamic pulse pressurisation resulting in a larger process zone due to frequent lowering of crack-tip stresses (modified from Zang et al.^1^). In (**b**), the transport of fracture-wall material (*rock disks* = natural proppants) towards the crack tip allows the local stress field to change and multiple branching fractures to develop, thereby forming an enlarged damage zone.

**Figure S2**. Crack growth rate in hydraulic fatigue.


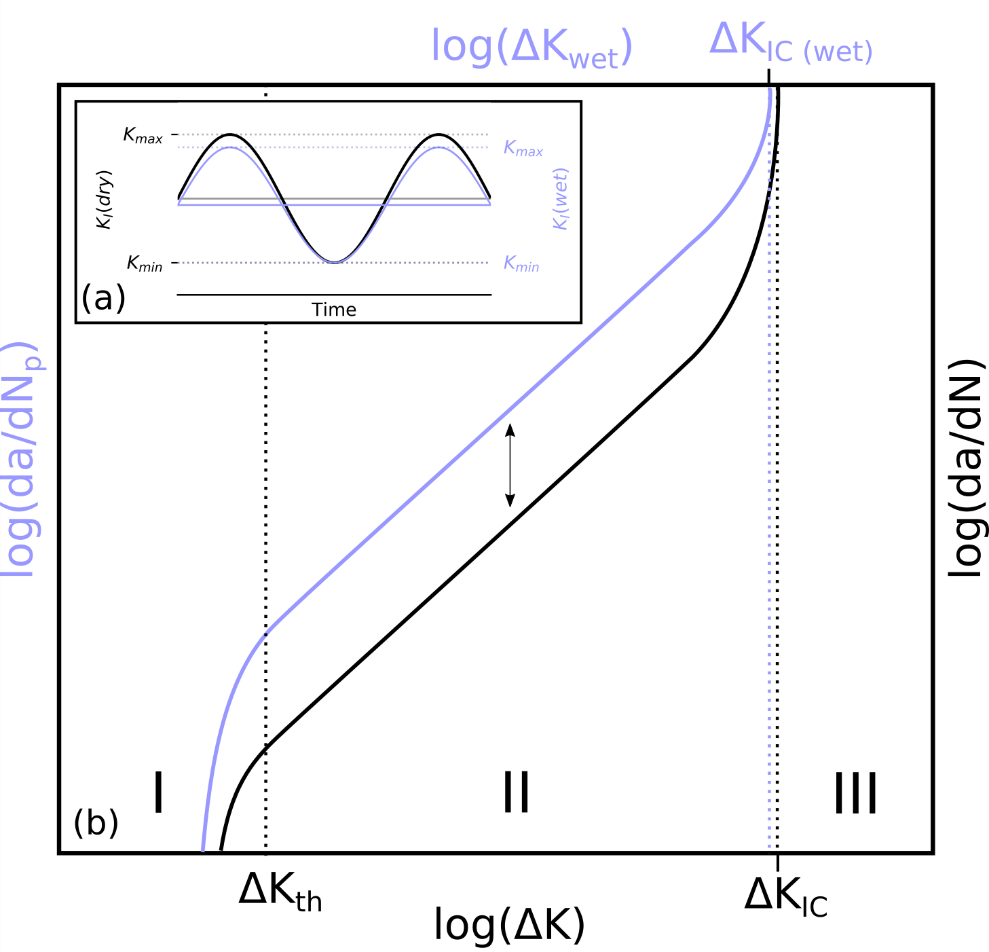


**Figure S2**. Fracture-mechanics formulation of mechanical fatigue (*black* curves) and hydraulic fatigue (*blue* curves). In (**a**), sinusoidal excitation of the stress-intensity factor (*K*) against time is shown for mechanical cycles (*black*) and hydraulic cycles (*blue*) with presumably lower stress-intensity factors. In (**b**), growth of fracture length per cycle (*da/dN*) is displayed against stress-intensity factor (*ΔK*) in a double-logarithmic plot. In the hydraulic-fatigue process, a fracture-growth rate is indicated that is higher compared with that in mechanical fatigue (**b**, *arrow*).

**Figure S3**. Seismic *b*-value determination in geothermal-injection tests across scales.


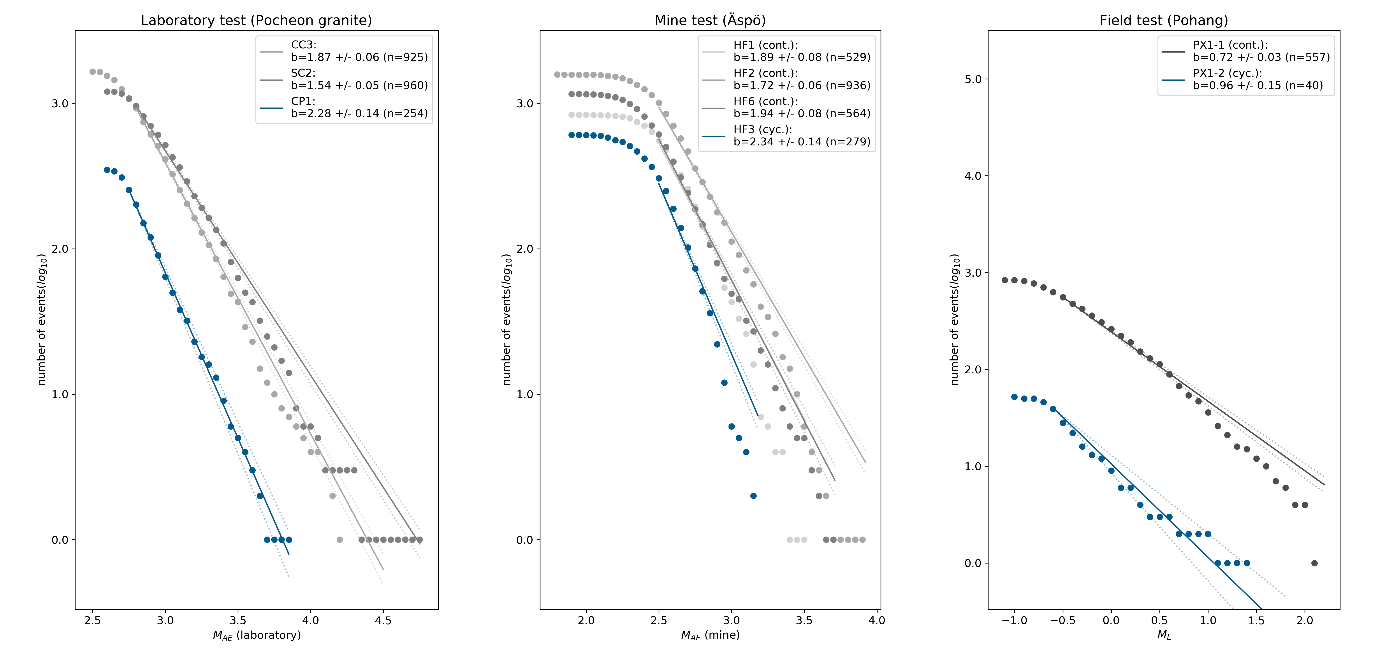


**Figure S3**. Seismic *b*-values determined via an analysis of acoustic-emission events in granitic rock at (**a**) laboratory scale and (**b**) mine scale in in-situ hydraulic-fracturing experiments. At field scale (**c**), micro-seismic events were used to compute magnitude-frequency distributions. The range of y-axis in (**c**) is adjusted to keep the ratio 1:1 (x:y) in all scales. Cyclic injection in *blue*; conventional, continuous injection experiments in *grey/black*.

**Figure S4. Additional laboratory pressure-controlled fracturing tests on Pocheon granite.**


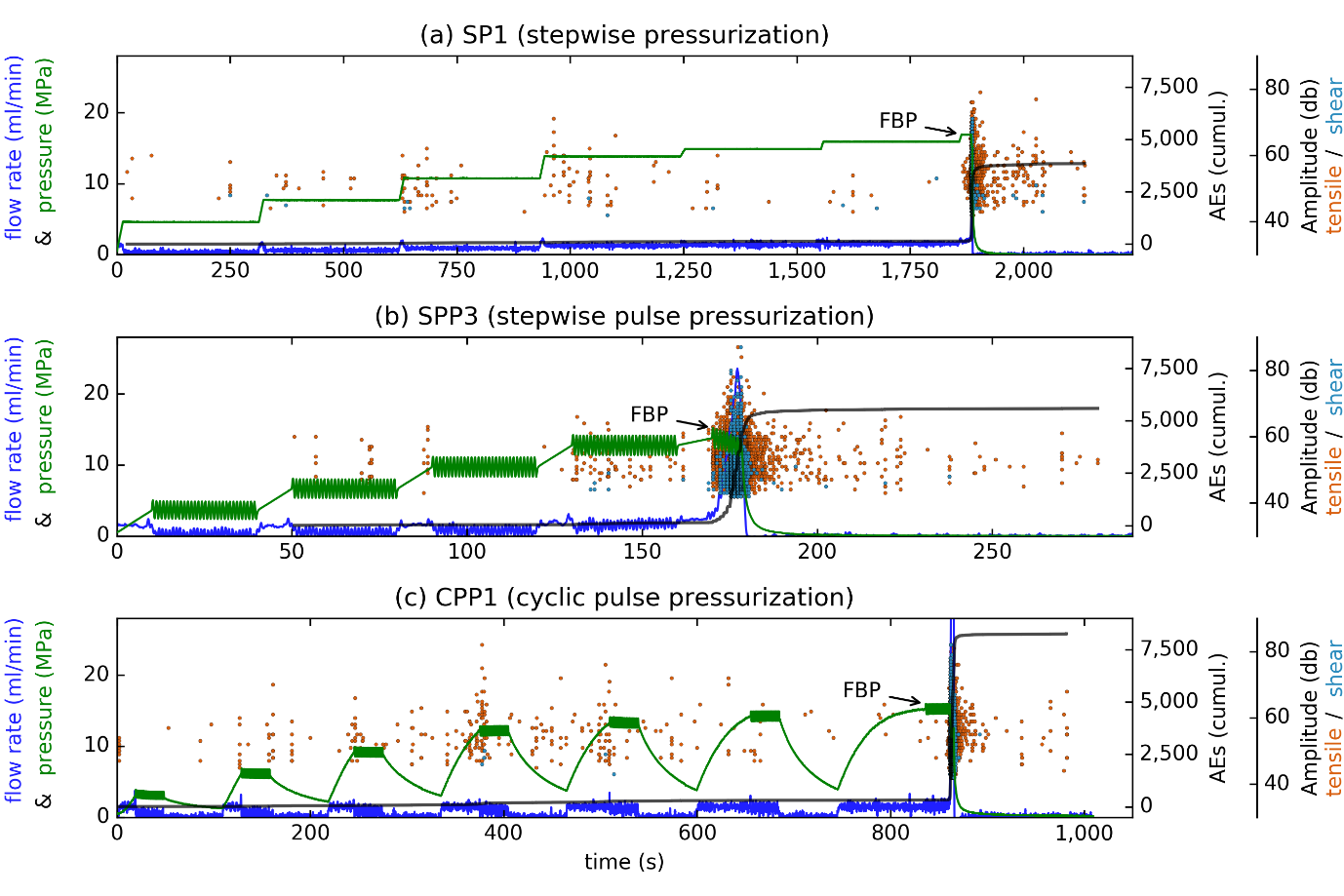


Figure S4 Laboratory hydraulic-fracturing results on true triaxially stressed Pocheon granite cubes under pressure control. (**a**) Stepwise (SP1), (**b**) stepwise-progressive (SPP3), and (**c**) cyclic-progressive pulse pressurisation (CPP1). *Red dots* indicate induced AE tensile failure; *light-blue dots* indicate induced AE shear failure.

**Figure S5. Additional mine hydraulic fracturing tests in two different rock types.**

**
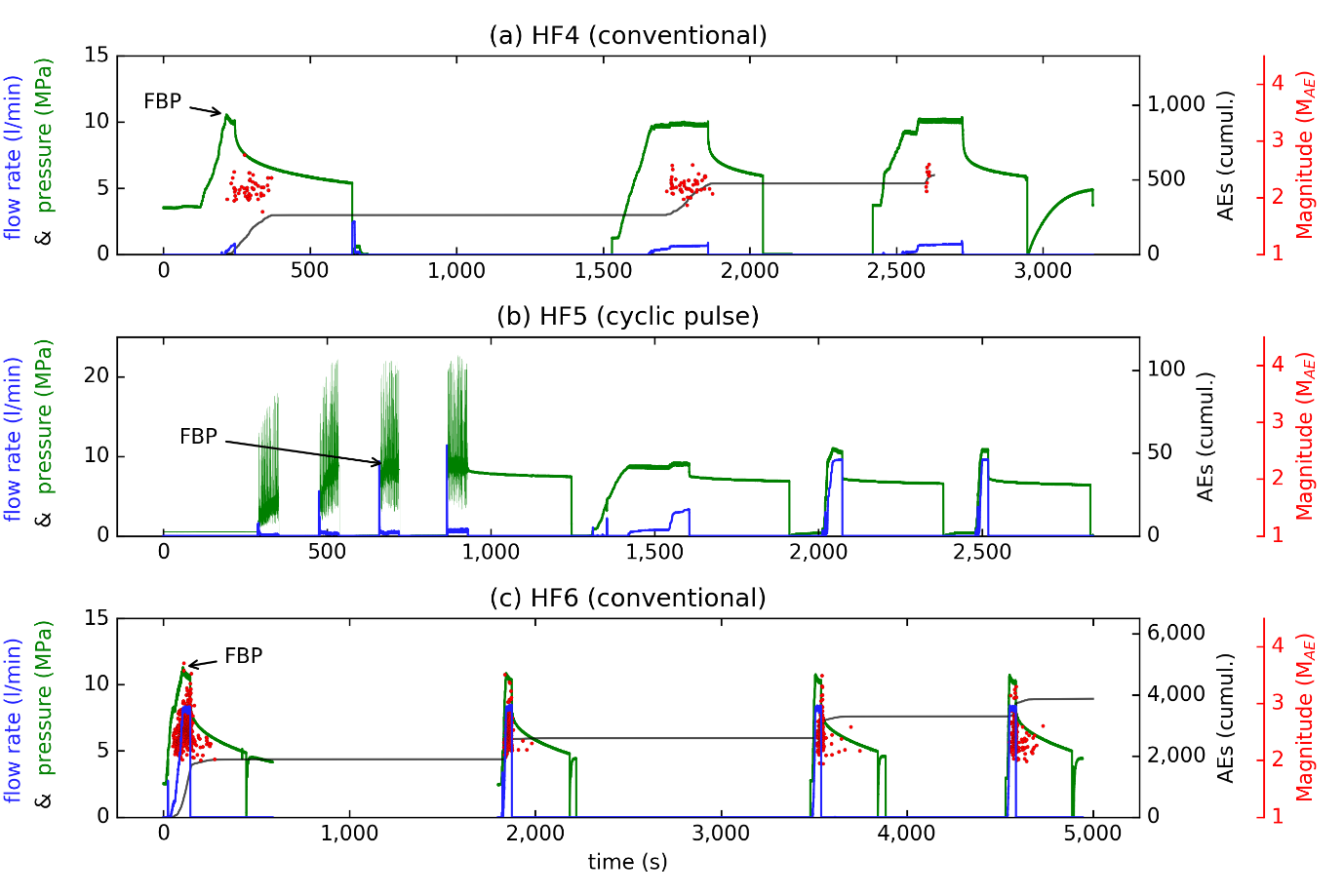
**

Figure S5 Mine hydraulic-fracturing results in two additional rock types. (**a**) Conventional fracturing in test HF4 and (**b**) cyclic-pulse fracturing in test HF5 with hydraulic hammer, both performed in fine-grained diorite-gabbro. (**c**) Conventional hydraulic fracturing (HF6) in fine-grained granite at six meter distance from tunnel wall.

**Figure S6. Breakdown pressure versus injection cycles in laboratory and mine tests**


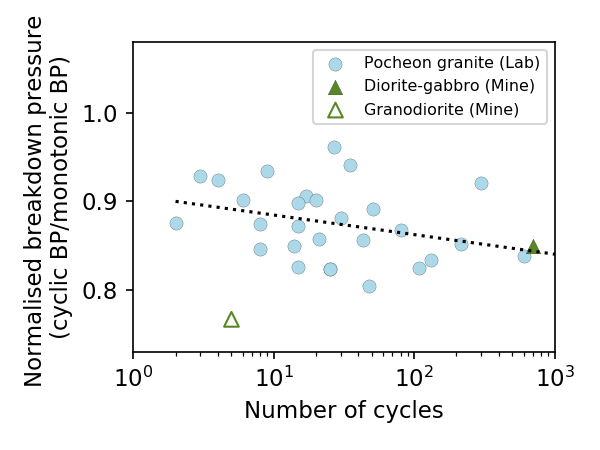


Figure S6 Cyclic breakdown pressure normalized to average conventional breakdown pressure as a function of log number injection cycles. *Dots* indicate laboratory tests on Pocheon granite. *Triangles* indicate mine tests. The 5-cycle fatigue test is performed in granodiorite (HF3), and the 700 cycles fatigue test with hydraulic hammer is performed in diorite gabbro (HF5).

(a)

**
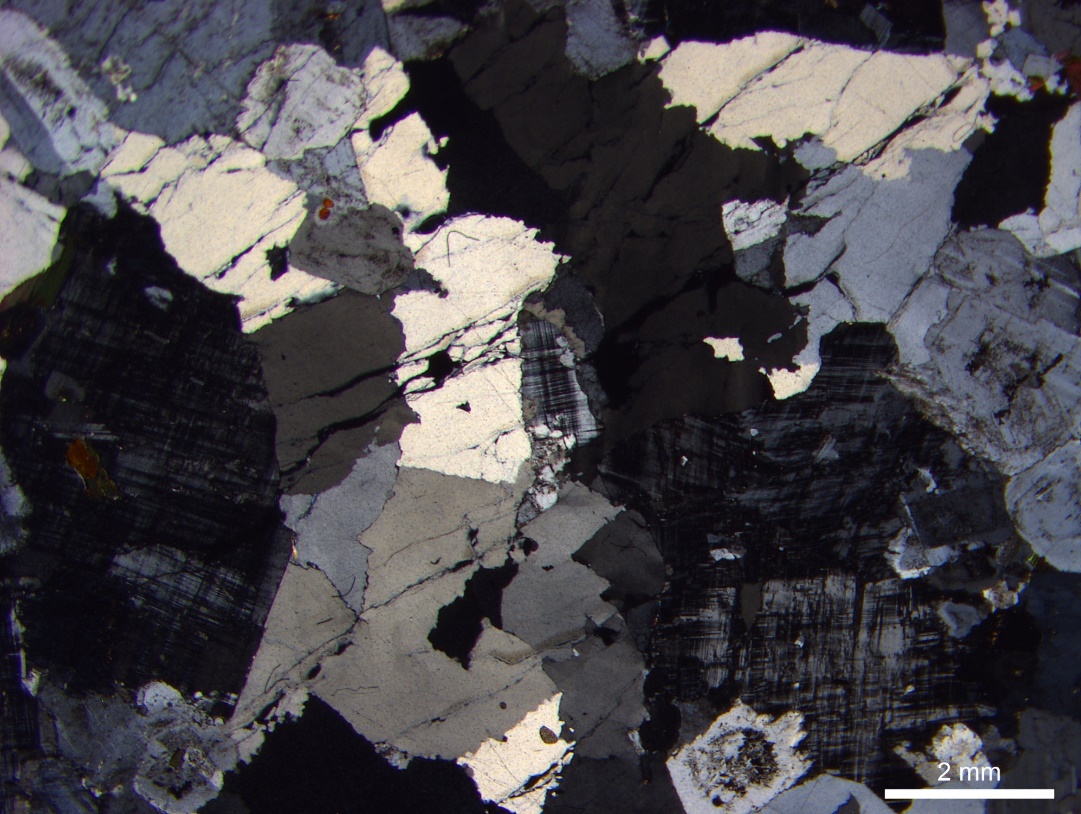
**

(b)

**Figure S7**. Fracture inspection after laboratory stepwise pulse pressurization test on Pocheon granite. (**a**) Microscopic photo collage with mineral identification along the hydraulic fracture path (borehole wall to the right). Mc-Microcline, Or-Orthoclase, Qtz-Quartz. (**b**) Selected enlarged section showing quartz fragments in hydraulic fractures. Scale bar is 2 mm in individual photographs.

**Table S8**. Full set of data used for computing energy terms of conventional and fatigue hydraulic fracturing across three scales: laboratory-, mine-, and field tests in granitic rock mass. Upper and lower bound values are given in brackets.

|  | True triaxial laboratory test  (Pocheon granite) | Mine test  (Äspö HRL,) | Field test  (Pohang EGS) |
| --- | --- | --- | --- |
| Dimension [m] | 0.1 x 0.1 x 0.1^[5]^ | 10 x10 x 3  (single experiment) | 1000 x 500 x 500 |
| Depth [m] | 100^*^ | 410^[2]^ | 4200^[8]^ |
| Principal stresses  (*σ_1_, σ_2_, σ_3_*) [MPa] | 6, 4, 3 ^[5]^ | 22, 12, 11 ^[2]^ | 139, 110, 82 ^[8]^ |
| Young’s modulus E [GPa] | 58 ^[5]^ | 60^[20]^ | 33 ^[8]^ |
| *E_Def_* [J] | 0.5 | 1.1e+06 | 59.56e+12 |
| Poisson ratio υ | [0.25, 0.31]^[5]^ | 0.25^[20]^ | 0.21 ^[21]^ |
| *G_IC_* (corrected) [MPa m^0.5^] | [21, 53] | [34, 77] | [512, 1159] |
| Fracture area [m^2^] | CC: 0.00448  SC: 0.00448  CP: 0.00225 | HF1: [37.0, 40.4]^[7]^  HF2: [41.0, 49.6]^[7]^  HF3: [28.4, 30.0]^[7]^  HF6: [36.3, 57.3]^[7]^ | Cont.: [37877,68882]  Cyc.: [231728, 407174] |
| *E_Frac_* [J] | CC: [0.09,0.24]  SC: [0.09,0.24]  CP: [0.05, 0.12] | HF1: [1.26e+03, 3.12e+03]  HF2: [1.40e+03, 3.83e+03]  HF3: [9.69e+02, 1.33e+03]  HF6: [1.24e+03, 4.44e+03] | Cont: [1.94e+07, 7.98e+07]  Cyc.: [1.19e+08, 4.72e+08] |
| *E_Hyd_* [J] | CC: 61.8  SC: 157.4  CP: 157.3 | HF1: 233.5e+03  HF2: 277.4e+03  HF3: 249.7e+03  HF6: 248.0e+03 | Cont: 91.0e+09  Cyc.: 28.5e+09 |
| Stress drop [MPa] | - | [0.1,1] | [0.1,1] |
| Shear modulus [GPa] | - | 24 | 14 |
| *E_Seis_* [J] | CC: [9.563e-05, 1.759e-03]  SC: [8.423e-05, 1.656e-03]  CP: [1.959e-05, 0.351e-03] | HF1: [1.22, 12.23]  HF2: [2.33, 23.25]  HF3: [0.75, 7.47]  HF6: [1.6, 16.0]  total: [5.99, 59.9] | Cont.: [3.74e+07, 3.74e+08]  Cyc.: [4.22e+06, 4.22e+07] |
| *E_Diss_* [J] | CC:[62.06,62.21]  SC:[157.66, 157.81]  CP:[157.68,157.75] | HF1: [1.33e+06, 1.33e+06]  HF2: [1.37e+06, 1.38e+06]  HF3: [1.35e+06, 1.35e+06]  HF6: [1.34e+06, 1.35e+06] | Cont.: [5.97e+13, 5.97e+13]  Cyc.: [5.96e+13, 5.96e+13] |
| $\frac{E_{Seis}}{E_{Hydr}}$ | CC:[1.547e-06, 2.846e-05]  SC:[5.351e-07, 1.052e-05]  CP:[1.245e-07, 2.231e-05] | HF1: [6.052e-06, 6.034e-05]  HF2: [9.639e-06, 9.653e-05]  HF3: [3.440e-06, 3.436e-05]  HF6: [7.419e-06, 7.415e-05] | Cont.: [4.110e-04,4.110e-03]  Cyc.: [1.481e-04, 1.481e-03] |
| $\frac{E_{Seis}}{E_{Hydr}+E_{Def}}$ | CC:[1.535e-06, 2.823e-05]  SC:[5.334e-07, 1.049e-05]  CP:[1.241e-07, 2.224e-05] | HF1: [1.058e-06, 1.055e-05]  HF2: [1.939e-06, 1.942e-05]  HF3: [6.370e-07, 6.363e-06]  HF6: [1.365e-06, 1.364e-05] | Cont.: [6.270e-07,6.270e-06]  Cyc.: [7.082e-08, 7.082e-07] |

*estimated from true triaxial stresses
